# Butterfly Effect in Cytarabine: Combined NMR-NQR Experiment, Solid-State Computational Modeling, Quantitative Structure-Property Relationships and Molecular Docking Study

**DOI:** 10.3390/ph17040445

**Published:** 2024-03-29

**Authors:** Jolanta Natalia Latosińska, Magdalena Latosińska, Janez Seliger, Veselko Žagar, Tomaž Apih

**Affiliations:** 1 Faculty of Physics, Adam Mickiewicz University, Uniwersytetu Poznańskiego 2, 61-614 Poznań, Poland; 2 “Jožef Stefan” Institute, Jamova 39, 1000 Ljubljana, Slovenia; 3 Faculty of Mathematics and Physics, University of Ljubljana, Jadranska 19, 1000 Ljubljana, Slovenia

**Keywords:** cytarabine, cytidine, NMR-NQR, binding mode, crystalline pattern, molecular docking

## Abstract

Cytarabine (Ara-C) is a synthetic isomer of cytidine that differs from cytidine and deoxycytidine only in the sugar. The use of arabinose instead of deoxyribose hinders the formation of phosphodiester linkages between pentoses, preventing the DNA chain from elongation and interrupting the DNA synthesis. The minor structural alteration (the inversion of hydroxyl at the 2′ positions of the sugar) leads to change of the biological activity from anti-depressant and DNA/RNA block builder to powerful anti-cancer. Our study aimed to determine the molecular nature of this phenomenon. Three ^1^H-^14^N NMR-NQR experimental techniques, followed by solid-state computational modelling (Quantum Theory of Atoms in Molecules, Reduced Density Gradient and 3D Hirshfeld surfaces), Quantitative Structure–Property Relationships, Spackman’s Hirshfeld surfaces and Molecular Docking were used. Multifaceted analysis—combining experiments, computational modeling and molecular docking—provides deep insight into three-dimensional packing at the atomic and molecular levels, but is challenging. A spectrum with nine lines indicating the existence of three chemically inequivalent nitrogen sites in the Ara-C molecule was recorded, and the lines were assigned to them. The influence of the structural alteration on the NQR parameters was modeled in the solid (GGA/RPBE). For the comprehensive description of the nature of these interactions several factors were considered, including relative reactivity and the involvement of heavy atoms in various non-covalent interactions. The binding modes in the solid state and complex with dCK were investigated using the novel approaches: radial plots, heatmaps and root-mean-square deviation of the binding mode. We identified the intramolecular OH···O hydrogen bond as the key factor responsible for forcing the glycone conformation and strengthening NH···O bonds with Gln97, Asp133 and Ara128, and stacking with Phe137. The titular butterfly effect is associated with both the inversion and the presence of this intramolecular hydrogen bond. Our study elucidates the differences in the binding modes of Ara-C and cytidine, which should guide the design of more potent anti-cancer and anti-viral analogues.

## 1. Introduction

**Figure 1:**
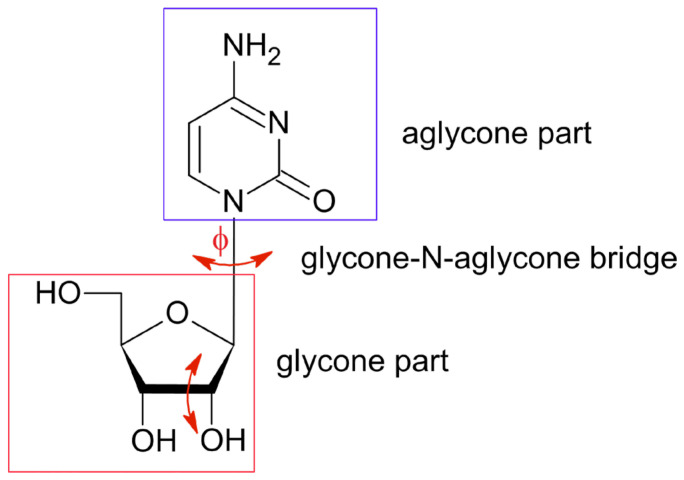
Molecular structure of nucleoside with a sugar group (glycone) bonded to a non-sugar (aglycone) via glycone-N-aglycone bridge (β-N1-glycosidic bond); ϕ—the rotation angle C(2)N(1)CO indicated by the upper red arrow. The lower red arrow indicates the difference in the glycone part: D-arabinose vs. ribose).

Recent drug development efforts have focused on the search for therapeutic agents that can effectively treat diseases associated with high global mortality: cancer and viral diseases. The class of compounds that holds significant importance is the nucleoside analogues, synthetic forms of natural nucleosides, nucleotides and bases that have been chemically modified. The naturally occurring nucleosides are composed of an aglycone moiety (a pyrimidine- or purine-derived base) and a glycone moiety (sugar: β-D-ribofuranose in the RNA or 2′-deoxy-β-D-ribofuranose in the DNA), Figure 1.

Nucleoside analogues with a modified aglycone part constitute a starting point for the synthesis of numerous chemotherapeutics, which possess significant binding affinities to both DNA and RNA. They act as competitive inhibitors of viral DNA or RNA synthesis, and when incorporated into the growing nucleic acid chain they cause premature termination of replication. After phosphorylation, their activity increases due to the presence of an additional fragment, which easily interacts with enzymes involved in nucleic acid synthesis. Thus, many of them are used as prodrugs. Both mechanisms make nucleoside analogues effective against viral infections (*HIV*, *Hepatitis B* and *Herpesvirus*) and in the treatment of certain types of cancer (especially hematological). Therefore, they constitute an important class of pharmaceutical active pharmaceutical ingredients (APIs), widely used in the drugs development.

**Figure 2:**
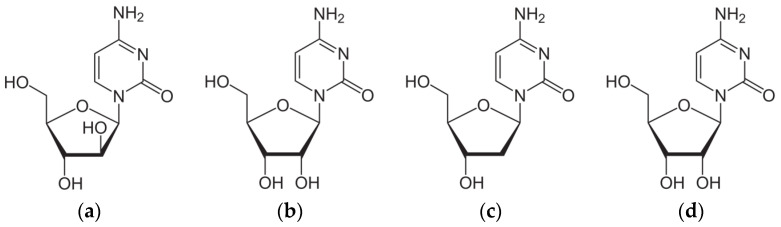

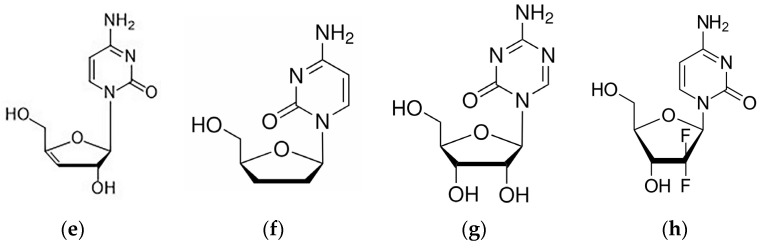
Structural formula of (**a**) Cytarabine (Cytosine arabinoside; Cytosine-1-β-D-arabinofuranoside; Ara-C) and closely related compounds (**b**) Cytidine (CTP), (**c**) 2′-deoxycytidine (dCTP), (**d**) 3′-deoxycytidine, (**e**) 3′-deoxy-3′,4′-didehydrocytidine, (**f**) 2′,3′-dideoxycytidine (ddCTP, Zalcitabine), (**g**) 5-azacytidine (5-AzaC, Vidaza) and (**h**) Gemcitabine (2′, 2′-difluoro-2′deoxycytidine, dFdC, Gem/Gemzar).

The isolation of a number of unique nucleosides including spongothymidine (3-β-D-arabofuranosylthymine) and spongouridine (3-β-D-arabofuranosyluracil) from the Caribbean sponge *Cryptotethya crypta* (now *Tectitethya crypta*) by Bergmann et al. [1], and the observation that these compounds can act as terminators of the DNA synthesis chain, initiated research on the new and separate class of nucleotides with modified glycone part: arabinosides. The possibility that they have a role as anti-viral, anti-cancer as well as anti-cellular senescence drug has sparked interest in them. In 1959, Walwick, Roberts and Dekkerin [2] synthesized Cytarabine, (Cytosine arabinoside; Cytosine-1-β-D-arabinofuranoside; Ara-C), a synthetic isomer of cytidine, that differs from cytidine and deoxycytidine, only in the sugar (D-arabinose instead of ribose and deoxyribose, respectively), Figure 2.

The constant interest in Ara-C results from its specific mode of action, the mechanism of which remains unclear despite the passage of time. Ara-C is known to be transported into the cell primarily by Human Equilibrative Nucleoside Transporter 1 (hENT-1). Activation of Ara-C in cells occurs as a result of de novo synthesis of 5′-mono-, di-, and triphosphate derivatives throughout the sequential action of nucleoside-phosphate kinases: deoxycytidine kinase (dCK), deoxycytidine monophosphate kinase (dCMK), and nucleoside diphosphate kinase (NDPK) [3]. dCK plays an important role in preventing Ara-C from being converted to an inactive metabolite called uracil arabinoside (Uracil-1-β-D-arabinofuranoside) by cytidine deaminase, which occurs by phosphorylating Ara-C. After being rapidly converted to arabinonucleoside triphosphate (Ara-C-CTP), it becomes a substrate of DNA polymerases α, β, γ and η [4]. Ara-C-CTP hinders DNA polymerases α and η, the enzyme responsible for initiating DNA replication, by outcompeting the natural substrate deoxycytidine triphosphate (dCTN-CTP). Nonetheless, it merely acts as a modest competitive inhibitor of this enzyme. Another factor has been shown to hinder DNA synthesis, namely the incorporation of Ara-C into DNA. The use of arabinose instead of deoxyribose hinders the formation of phosphodiester linkages between pentoses, preventing the DNA chain from elongation. As a result, the DNA synthesis process is interrupted, the cell cycle from G1 to the S phase is blocked and apoptosis occurs. The use of a non-functional nucleoside to inhibit DNA strand extension is a strategy used in other chemotherapeutics of natural origins (e.g., adenine arabinose or azidothymidine), with only Ara-C having strong anti-cancer properties, while the remaining analogues have anti-viral properties. Although Ara-C has some anti-viral activity against *Herpesvirus* (HHV) [5] and *Vaccinia Virus* (VV) [6], it is not highly selective and causes serious side effects.

Ara-C is highly effective against hematologic malignancies (e.g., acute lymphocytic leukemia, acute/chronic myeloid leukemia, acute promyelocytic leukemia, anaplastic large cell lymphoma, Burkitt lymphoma, Langerhans cell histiocytosis, leptomeningeal carcinoma and non-Hodgkin’s lymphoma) [7,8] and its efficacy against carcinomas is limited to sarcomas (Nakahara-Fukuoka sarcoma, reticulum cell sarcoma, ascites sarcoma-180), adenocarcinomas (adenocarcinoma-755, spontaneous mammary adenocarcinoma) [9,10] and Ehrlich ascites carcinoma [11], among others, by the method of administration and side effects (chronic toxicity to internal organs including cerebrum). Efforts to improve its efficacy against the so-called “civilization diseases”, through drug synergism, targets modification or allosteric modulation, are ongoing. Ara-C exhibit remarkable synergistic effect with other anti-cancer drugs including Daunorubicin, Vinblastine and cis-diaminodichloroplatin (II) (CDDP). Recently, it has been discovered that combining Ara-C with daunorubicin or anthracyclines in liposomal formulations leads to the significant positive outcomes of about 60% [12,13] or 80% [14], respectively. Low-dose cytarabine combined with Venetoclax and 5-azacitidine achieved remission rates of 57% while combined with Decitabine (5-aza-2′-deoxycytidine) 75% for newly diagnosed older or unfit AML patients [15,16], respectively. The new meta-analysis carried out in [17] has confirmed that the addition of cytarabine to the therapeutic regimen of newly diagnosed patients with primary central nervous system lymphoma (PCNSL) patients is highly beneficial. In Europe and the United States, multiple courses of high-dose Ara-C are used as standard consolidation treatment for AML patients who have achieved complete remission [18]. This therapeutic protocol results in higher long-term survival especially for patients in the intermediate-risk group (2022 ELN risk stratification) [19]. Therefore, Ara-C is one of the few anti-cancer drugs that have been used in nearly unmodified treatment protocols for more than 50 years. Its presence on the World Health Organization’s eEML Model List testifies to the importance it holds.

Surprisingly, Ara-C and naturally occurring cytidine, that differ only in the orientation of a single hydroxyl group in the 2′ position (as shown in Figure 2a,b) exhibit completely different biological activities. Cytidine is used as a glutamatergic anti-depressant drug to manage neuropsychiatric deficits associated with cerebrovascular diseases by controlling neuronal-glial glutamate cycling. Its active form, 5′-triphosphate CTP, takes part in the synthesis of ribonucleic acid (RNA) as a building block or as an activating group in the synthesis of lipids such as lecithin, cephalin and cardiolipin. Therefore, the small local structural change limited to the glycone moiety has significant consequences for biological activity.

The aim of our study is to explain how much the hydroxyl inversion, optimization or change of a substituent within glycone moiety affects the distribution of electron density the molecule, and thus the ability to form intermolecular bonds, which determines its biological activity.

For this purpose, we have chosen a highly sensitive technique that is able to detect local changes, even in places that are far away from any structural changes. The physical property describing the charge distribution in molecules and their charge related properties is electrostatic potential, which is directly proportional to the magnitude of the charge and inversely proportional to the distance from the charge. However, its variability between individual molecular environments is relatively small and subtle changes are difficult to assess. The set of the second-order derivatives of the electrostatic potential constituting the Electric Field Gradient (EFG) tensor, is very sensitive even to minor changes in the electron density distribution at the nucleus. The substituent effects or molecular motions result in the changes in the electrostatic potential reflected by the EFG tensor and observables-NQR frequencies. Since the components of the EFG scale with 1/r^3^, where r is the distance from the nucleus, they are highly sensitive primarily to the changes in the immediate vicinity of the nucleus, and slightly weaker to the changes relatively distant from the nucleus. By probing the EFG it is possible to gain insight into the electron density distribution throughout the molecule. The experimentally detected or theoretically calculated components of the EFG tensor provide a description of the nearest nuclear vicinity. However, from an experimental point of view, it is not easy task and demand the use of three ^1^H-^14^N NMR-NQR (Nuclear Magnetic Resonance–Nuclear Quadrupole Resonance) techniques, namely multiple frequency sweeps, Larmor frequency scanning, and the two-frequency irradiation. When supplemented with the solid-state computational modelling (Quantum Theory of Atoms in Molecules [20,21], Reduced Density Gradient [22] and 3D Hirshfeld surfaces [23]), and Quantum Structure–Property Relationship [24] deliver deep insight into three-dimensional packing and reactivity at the atomic and molecular levels.

These methods shed some light on the pattern of intra- and intermolecular interactions in solid Ara-C, cytidine, and some closely related compounds lacking at least one -OH group (2′-deoxycytidine, 3′-deoxycytidine, 3′-deoxy-3′,4′-didehydrocytidine, 5-azacytidine, Zalcitabine, Decitabine or Gemcitabine). Ara-C and cytidine differ only slightly, so one would expect their interaction patterns to be similar and the differences to be no greater than those observed in the polymorphic forms. However, a slight structural alteration, the inversion of hydroxyl group at the 2′-position of the sugar, leads to change of the biological activity from antidepressant and DNA/RNA block builder (cytidine) to powerful anti-cancer (Ara-C). We have discovered that above mentioned modification causes significant changes in the electron density distribution and binding mode, clearly visible in the NQR spectrum. The use of molecular docking enabled us to examine and explain the binding of Ara-C and closely related compounds to dCK. We identified the intramolecular OH···O hydrogen bond as the key factor responsible for forcing the glycone conformation and strengthening NH···O bonds with Gln97, Asp133 and Ara128, and stacking with Phe137. The title butterfly effect is associated with its presence.

However, our aim was not only to identify differences in ligand binding but also to develop a method for comparing them in the solid state and in protein–ligand complexes. To date, no thorough comparative analysis of binding modes in solid-state and protein–ligand complexes upon changes in ligands has been conducted. Therefore, we have developed new methods for quantitative and qualitative comparison of binding modes. Heat maps, that are commonly used to compare amino acid sequences in proteins, can help to visualize and classify differences in binding modes in both the solid state and protein–ligand complexes. A new parameter, RMSD_BM (the root mean square distance between binding modes), has been introduced to describe the relative distance between the binding modes in both cases using numerical values. It has been demonstrated that modifications to the glycone moiety have an impact on the distribution of electron density within the aglycone moiety. This, in turn, affects its binding capacity in both the solid state and protein–ligand complex. By correlating these findings with the relative ligand reactivity parameter recently introduced by us, it is possible to screen the ligands for their potential biological activity. The glycone moiety plays a crucial role in the ligands studied as it significantly influences the electron density distribution within aglycone moiety. The comparison of the selected ligands, namely Ara-C (anti-leukemic), cytidine (anti-depressant and RNA component), Gemcitabine (anti-cancer), Zalcitabine (anti-retroviral) and Decitabine (anti-leukemic and anti-virial) performed in this paper illustrates the usefulness of the new methods quite well. The methods proposed for comparing ligand reactivity, solid-state and protein-ligand binding modes show promise for virtual screening ligands and effective search for drug improvements.

## 2. Results and Discussion

### 2.1. ^1^H-^14^N NQDR Spectrum

We used Larmor frequency scanning [25] and multiple frequency sweep [26] as the first ^14^N NQR frequency localization techniques. Measurements were performed at room temperature (295 K).

The proton polarization period was set to 30 s and the relaxation period was set to 0.5 s. We used linear frequency sweeps in the range from 1.6 MHz to 3.5 MHz. The duration of a single cycle was set to 10 ms. The average amplitude of the RF magnetic field during the sweep was approximately 2 mT. The Larmor proton frequency scan was performed in 10 kHz steps, repeating it four times and averaging at each step. By this technique, the low-frequency part of the ^14^N NQR spectrum of Ara-C was detected. In the next step, by varying the frequency limits of the sweeps, we located the higher ^14^N NQR frequencies ν_+_ and ν_−_. In order to increase the resolution to about ±5 kHz, we used as the final step the two-frequency irradiation technique. The two rf magnetic fields were applied as a series of repetitive pulses with the pulse length of 1 ms. The amplitudes of the two rf magnetic fields were set to 0.2 mT.

**Figure 3:**
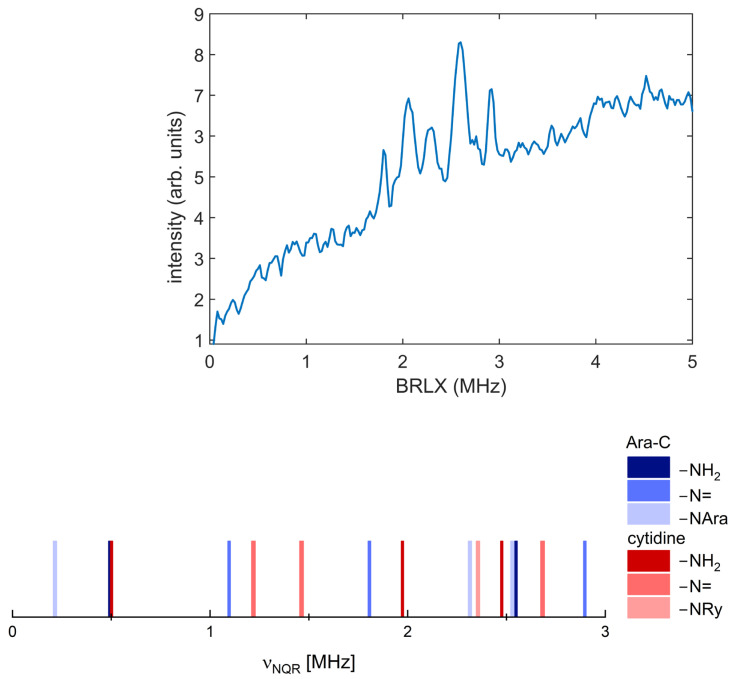
The experimentally determined ^14^N NQR spectrum in Ara-C (**top**) and positions of the resonance lines in the spectra of Ara-C and reference cytidine.

The ^14^N NQR spectrum and position of the lines are shown in Figure 3, and the frequencies and e^2^qQ/h and η calculated using Equation (3) are collected in Table 1.

So far, only a few compounds structurally similar to Ara-C have been investigated. The few spectra recorded for them are usually incomplete (e.g., cytidine and 5-azacytidine, Table 1). While the detection of frequencies for the –NH_2_ group did not pose any particular difficulties, the remaining frequencies are often missing (cytidine and 5-azacytidine [27]), Table 1. Nonetheless, the NQR spectrum of Ara-C differs significantly from that of structurally similar cytidine, Figure 3.

The resonance lines in NQR spectrum assigned to –N= and >N–sugar are “clean”, i.e., they are normally broadened, with close full width at half maximum (FWHM), implying no structural disorder. The absence of multiplicity implies the presence of equivalent molecules per unit cell, which is generally consistent with the X-ray results [30].

The surroundings of the three nitrogen nuclei in the Ara-C molecule show significant differences due to hybridization and substituent effects, making them chemically inequivalent. It is highly likely that nitrogen signals can be correctly assigned solely based on the NQR spectra, due to the different e^2^Qq/h and η values for each of them. In Ara-C molecule, the N(1) nitrogen frequencies can be readily distinguished from others due to the strong proton–nitrogen interaction, which affects the double resonance spectra. Therefore, they can be assigned to –NH_2_, for which level crossing and solid effects are usually easy observed. However, the –NH_2_ resonance lines appear to be doublets despite the lack of disorder in X-ray structure. In most NQR studies, the quadrupole interaction is assumed to be steady, but certain molecular motions can strongly affect the quadrupolar interaction, leading to the EFG tensor fluctuations. The formation of doublets can be the result of molecular motions: hindered rotation of the –NH_2_ group around the NC axis (two site jumps) and/or proton-jumps (proton hopping) in hydrogen bonds. The former is usually activated at relatively high temperatures, while the latter occurs even below liquid nitrogen temperatures. The –NH_2_ group of Ara-C is involved in two N–H∙∙∙O hydrogen bonds, which are bent. The N∙∙∙O distances are remarkably short (2.983 and 3.048 Å). The proton transfer scores calculated for these bonds based on geometrical parameters are 7.45 and 8.40, respectively. Thus, the shorter one should be slightly more stable. However, at the higher temperatures, the hydrogen bond involving 3′-oxygen may be weakened due to the large amplitude motion of the sugar ring. This kind of effect was observed in 5-azacytidine and cytidine [27,28] as well as thymidine by ^2^H NMR [29].

The set of resonance lines 2.530, 2.315 and 0.215 MHz assigned to nitrogen N(3) are characterized by a low asymmetry parameter. There are two possible nitrogen sites in the Ara-C molecule, –N= and >N-sugar, to which this set of lines can be assigned. However, the nitrogen >N-sugar forms the glycone-N-aglycone bridge and participate in the three single N-C covalent bonds, Figure 1. Even though it is located close to the oxygen atom, whose lone pairs are adjacent to the π system, a relatively low asymmetry parameter is expected. Thus, this set of frequencies appears to correspond to >N-sugar nitrogen site. This is consistent with the fact that frequencies for this kind of nitrogen site can be detected easily using the combined multiple frequency sweeping and dual-frequency irradiation techniques.

Detecting the resonance lines originating from –N= nitrogen is a challenging task. This nitrogen participates only in two NC covalent bonds, which indicates a high value of the asymmetry parameter, similar to that for the –N= nitrogen of cytidine or cytosine. However, the addition of an extra nitrogen atom in 5-azacytidine reduces the asymmetry parameter because this nitrogen shares its lone pair with the π system, Table 1. The NQR frequencies for this nitrogen site can only be detected using the advanced Fast Field Cycling (FFC) relaxometry techniques.

Based on the experimental data, it seems that the two sets of resonance lines yielding in the lowest η should be assigned to the >N-sugar and –NH_2_ sites, while the third to the remaining –N=. Tentative assignments to individual sites are provided in Table 1.

**Figure 4:**
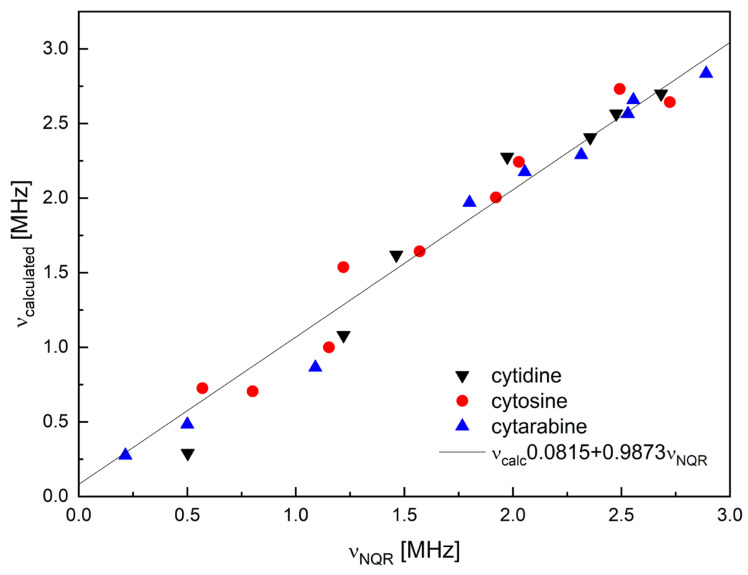
The correlation between the experimental and calculated (GGA/RPBE level, solid) NQR frequencies.

Quantum chemical calculations to verify the correctness of the frequency assignment were performed in different variants (single molecule, cluster and crystal). Modeling the NQR spectrum by assuming a single molecule is almost always too imprecise due to the neglect of the surroundings. The only advantage of this approach is that it does not require knowledge of the crystal structure, but at the cost of conformational searches that may not always yield a solid-state conformer. Two other approaches (cluster and solid) require a consistent, high-quality crystalline structure as input, but do not require conformational analysis, except in special cases such as structural or dynamic disorder. The Cambridge Structural Database (CSD) contains only one Ara-C crystalline structure [27]. Calculations were performed for this specific structure, having previously optimized the positions of the hydrogen atoms. We have also predicted the NQR spectrum for β-cytidine and 5-azacytidine, and for other compounds for which crystallographic structures are available. The results are listed in Table 2 and visualized in Figure 4.

Calculations performed at the GGA/RBPE level allow obtaining reasonable NQR parameters at a relatively low computational cost. Optimization of the proton positions is first step, especially important for the –NH_2_ group and results in a decrease in η and in an increase in |e^2^qQ/h|. Using other GGA functionals results in more scattered frequencies but the same assignment. Cluster calculations performed at the M062X/6-311+G(d,p) level additionally confirmed the correctness of frequency assignment. Moreover, the results of the calculations suggest that the spectrum for 5-azacytidine was previously misinterpreted [27], and the set of frequencies for the –N= site does not correspond to different nitrogen locations, but to a doublet. Furthermore, the NQR parameters indicate that the X-ray structure of 3′-deoxycytidine [33] is very unreliable (one of the inequivalent molecules has a defective geometry).

In general, NQR data suggest that –NH_2_ and to a lesser extent –N= form hydrogen bonds in solid state and thus should play a key role in intermolecular bond formation in Ara-C, cytidine and related compounds.

The –NH_2_ amine group is flexible and has lone pairs localized on the atoms adjacent to the π system. Thus, it is electron donating group, prone to forming strong N–H···O hydrogen bonds and activating the aromatic ring by increasing the electron density on the ring at the ortho and para positions. The oxygen =O substituted directly into the pyrazine ring at the C(4) position, is both σ electron withdrawing and strongly π electron donating and acts similarly to –NH_2_. Therefore, both are excellent π-donors and strongly affects –N(3)= and Ry. A comparison of the ^14^N NQR parameters modeled in the absence and presence of hydrogen bonds revealed the effect of hydrogen bond. The population of the π orbital, which is normal to the plane containing N–C and N–H bonds is higher than the population of their σ-orbitals. Thus, the Z axis of the EFG tensor at –NH_2_ is orientated along the π orbital. Any increase in the population of the N–H bond, due to the charge transfer from the donor electron pair in the hydrogen bond results in a decrease in e^2^Qq/h. But the N–H bond population is higher than that of N–C, hence the deviation from the axial symmetry increases, and consequently η increases. The fact that e^2^Qq/h is significantly lower, but η higher, for the amine nitrogen in the solid than in a single molecule supports the aforementioned argument and is consistent with other observations (e.g., imidazole).

The NQR parameters of –N(3)= correspond to the populations of the lone-pair orbital σ_LP_ and the nitrogen orbitals involved in the σ_NC_ and π bonds to carbon. Upon hydrogen bond formation, the EFG tensor at –N(3)= maintains its orientation. However, a decrease in σ_LP_ of –N(3)= leads to higher e^2^Qq/h and η values. The frequencies ν_0_, ν_+_, and ν_−_ are proportional to the populations of the nitrogen atom bonds and both NQR parameters. As a result, the NQR frequencies have changed slightly. The solid-state shift of the NQR parameters for nitrogen –N(3)= is rather minor, with η well reproduced and e^2^Qq/h is only slightly exaggerated. The latter suggests that –N(3)= is involved in weak intermolecular interactions in the solid state.

A comparison of the NQR parameters for Ara-C, cytidine and cytosine shows an increase in e^2^Qq/h and a decrease in η, after adding glycone residue (ribose) or changing it from ribose to arabinose regardless of the nitrogen site. Despite a minor structural modification, the variations in NQR parameters that reflect these changes are readily apparent. However, it is uncertain whether the observed differences are a result of substitution or a different mode of binding.

### 2.2. Intermolecular Interactions Pattern in Crystal

#### 2.2.1. Binding Motifs

**Figure 5:**
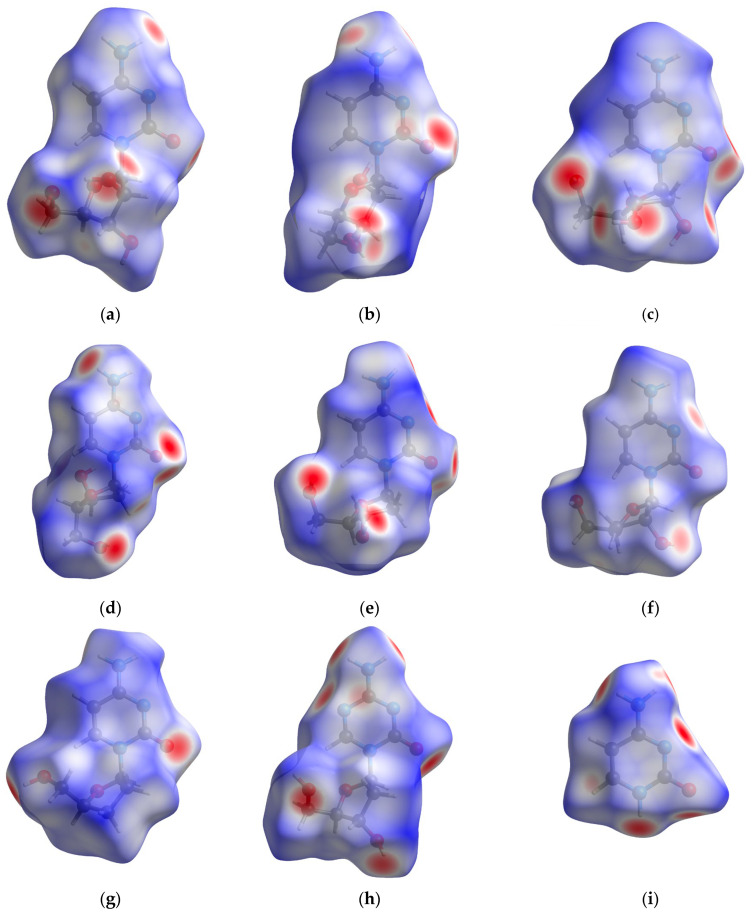
The intermolecular interactions visualized in the d_norm_ surface projected over 3D Hirshfeld Surfaces in a red-white-blue scheme (red: negative, white: zero, blue: positive), with red representing small range contacts such as hydrogen bonds, white representing contacts of approximately van der Waals radii, and blue representing the remaining, considerably longer, connections: (**a**) Ara-C, (**b**) α-cytidine, (**c**) β-cytidine, (**d**) 2′-deoxycytidine (αIrm) (**e**) 2′deoxycytidine (β form), (**f**) 3′-deoxycytidine, (**g**) 2′,3′-dideoxycytidine (Zalcitabine), (**h**) 5-aza-2′-deoxycytidine (Decitabine) and (**i**) cytosine.

Hydrogen bonds are the most common non-covalent molecular binding motifs in drug structures. Although the Etter rule [36,37] states that the molecule will attempt to form as many hydrogen bonds as possible, in practice some of them may not be feasible (e.g., due to a specific conformation or involvement in the intramolecular interactions). Ara-C containing amine, oxygen and hydroxy moieties is highly prone to form them in solid and in protein–ligand complexes. Even in solid, Ara-C and cytidine structures differ only slightly, thus, their interaction patterns are anticipated to be similar. Differences no greater than those typically observed in polymorphic forms are expected. A comparison with 2′-deoxycytidine, 3′-deoxycytidine and 2′,3′-dideoxycytidine, which have at least one less –OH group, and the inclusion of different polymorphic forms can provide a reliable reference point. Unfortunately, the 5-azacytidine or Gemcitabine solid-state structures has not yet been resolved. The analysis of 3D Hirshfeld Surfaces (3D HS), Figure 5, provides deep insight into the homo- and hetero-nuclear intermolecular contacts, Table 3.

**Figure 6:**
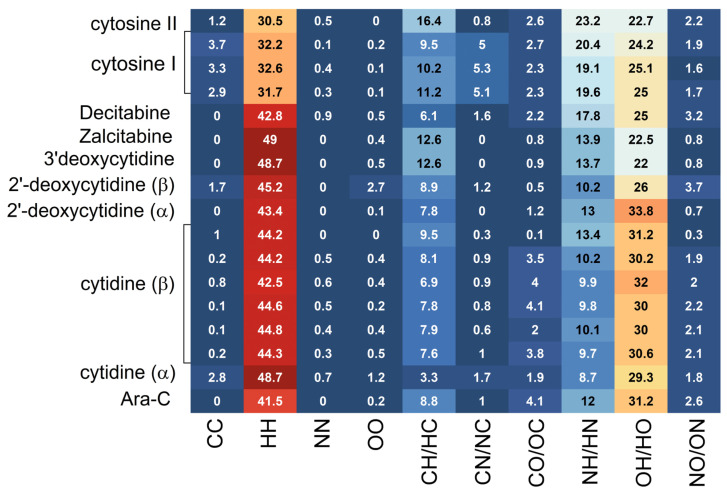
Heatmap visualizing the percentage contributions to the 3D Hirshfeld surface area calculated for each pair of species. The red-yellow-blue scheme, with dark red indicating strong interactions and dark blue indicating very weak ones was used.

Heatmap, visualizing the percentage contributions to the 3D Hirshfeld surface area calculated for each pair of species listed in Table 3, is shown in Figure 6

**Figure 7:**
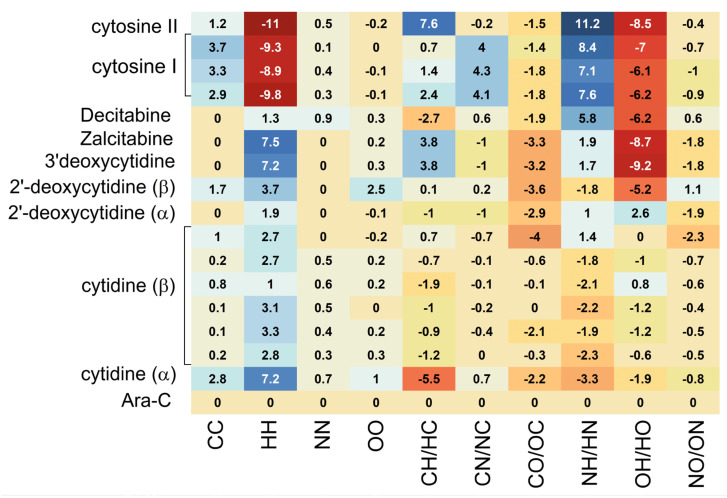
Difference heat map visualizing relative percentage contributions to the 3D Hirshfeld surface area calculated for each pair of species (Ara-C is a reference). The difference heat map visualizes the differences in the percentages in a red-yellow-blue scheme, where red indicates an increase and blue indicates a decrease.

Due to the identical atomic composition the same types of contacts occur in all systems, Table 3, Figure 6. About 50–70% of the percentage contributions comes from HH and OH/HO contacts. The CH/HC and NH/HN have much smaller contributions of about 8–16% and 10–20%, respectively. The remaining homonuclear (CC, NN and OO) or heteronuclear (NO/ON, NC/CN and CO/OC) contributions are negligibly small. Moreover, the dominant contacts are consistent across all studied ligands, with only their percentages varying. However, after sugar substitution, the amount of NH/HN and CH/HC interactions decreases, while OH/HO and HH increases, Figure 7.

As can be seen from Figure 7, the effect of the inversion of the hydroxyl group in the 2′ position of the sugar is subtle, and its detection strongly depends on the quality of the structure. The poorer the quality of the structure, the harder it is to notice. Moreover, polymorphic form matters.

The Euclidean distance (ED) and root mean square deviation (RMSD) showing the global differences between the percentages in the 3D Hirshfeld of all contacts for Ara-C and the compounds studied have the lowest values for β-cytidine and 2′-deoxycytidine, and the highest for cytosine, Table 4.

The values of the enrichment ratio, E_XY_, of the main intermolecular contacts, which reveals privileged (E_XY_ > 1) and disfavored (E_XY_ < 1) contacts between every two atomic species, X and Y, are collected in Table 5.

H atoms provide up to 70% of the molecular surface in investigated compounds, whereas the other contributing components C, O, and N generate much smaller but very similar percentages of the molecular surface—from about 6 to 18%, Table 5.

The percentages of the molecular surface of the crystalline structures of the nucleosides studied, are close (the differences do not exceed 3–5%), while those for cytosine differ by as much as 10%. The Euclidean distance (ED), Table 6, showing the difference between the enrichment ratios in Ara-C and studied compounds, takes the lowest values for 2′-deoxycytidine, Zalcitabine and β-cytidine, and the highest for α-cytidine and 3′-deoxycytidine. Thus, the privileged and disfavored contacts between every two atomic species are in 2′-deoxycytidine and β-cytidine similar to Ara-C. It should be noted that they differ significantly for each pair of polymorphic forms.

Detailed inspection shows that Ara-C resembles the β-form of cytidine in terms of the interactions pattern. Among C···C and H···C interactions that compete in the crystal structure, only C⋯H contacts are preferred, but their enrichment ratio, E_CH_, in Ara-C, cytidine and cytosine is smaller than unity, because H are involved in hydrogen bonds. In 2′-deoxycytidine, E_CH_ > 1, which suggests that H participates in hydrogen bonds to a lesser extent. The C···C contacts are significantly impoverished in all studies structures, E_CC_ ≈ 0 or E_CC_ > 2 (due to division by small number), so their participation in π···π stacking is electrostatically unfavorable. A very high E_CH_/E_CC_ in Ara-C and β-cytidine suggests the classification of the packing motifs as close and herringbone type. The H···H contacts appear with an enrichment ratio slightly smaller than unity (E_HH_ ≈ 0.9) due to their strong competition with the hydrogen acceptors H···N (E_NH_ > 1.0) and H···O (E_OH_ > 1.0). The O···H/H···O contacts show enrichment values (E_OH_ ≈ 1.3) because they are favored in the crystal packing, and, notably, the O=C oxygen atoms form intermolecular hydrogen bonds of the type O–H···O and N–H···O. Therefore, the O···O and N···O contacts are significantly impoverished. The N···H/H···N contacts show enrichment values higher than unity, and, thus, are favored and the existence of the O–H···N hydrogen bonds is raised. In general, in Ara-C, α-cytidine and cytosine the favored interaction partners for N, whose contribution to the 3D HS molecular surface is about 7, 13 and 6%, respectively, are C and H species, while O, followed by N are disfavored. In β-cytidine and 2′-deoxycytidine the favored interactions partner for N, whose contribution to the 3D HS molecular surface is about 6%, is only H species, while C, followed by O and N are disfavored. In all structures the favored interaction partners for O are H, which suggests its participation in many hydrogen bonds.

**Figure 8:**
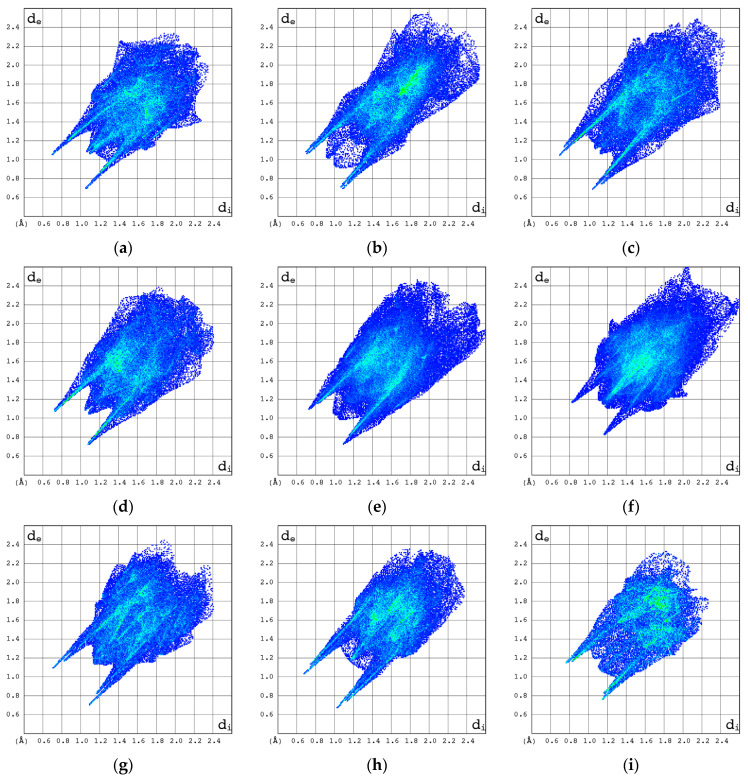
The total 2D molecular fingerprints: (**a**) Ara-C, (**b**) α-cytidine, (**c**) β-cytidine, (**d**) 2′-deoxycytidineI form) (**e**) 2′-deoxycytidine (β form), (**f**) 3′-deoxycytidine, (**g**) 2′,3′-dideoxycytidine (Zalcitabine), (**h**) 5-aza-2′-deoxycytidine (Decitabine) and (**i**) cytosine. (The white color represents the contacts nearer to sum of the vdW radii, while red and blue color indicates shorter and longer contacts).

The participation of C, N, and O atoms in the individual intermolecular hydrogen bonds can be identified with the help of the 2D molecular fingerprints calculated based on 3D Hirschfeld surfaces. As follows from Figure 8, they differ significantly, in both shape and size for all the compounds studied.

**Figure 9:**
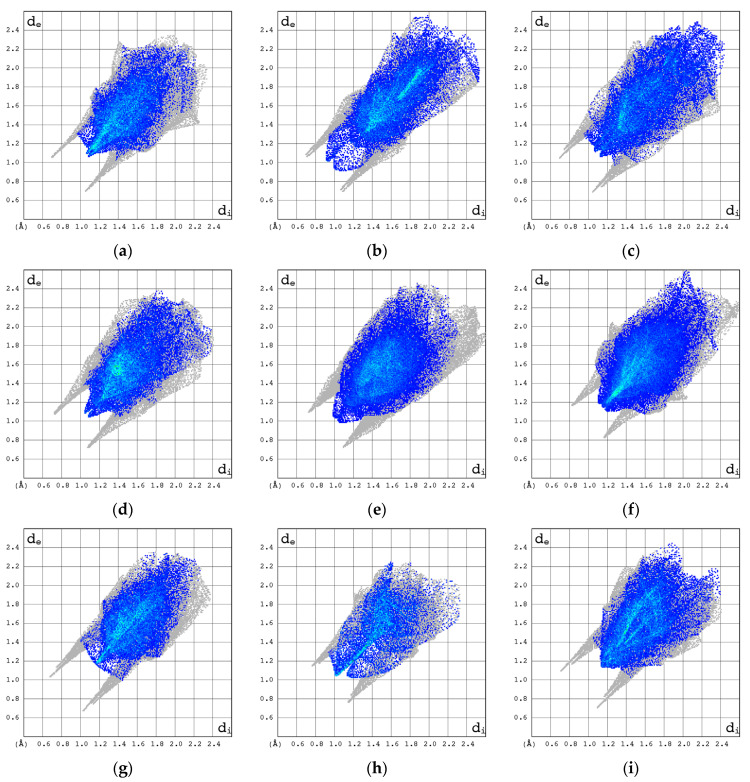
The 2D molecular fingerprints limited to HH interactions (**a**) Ara-C, (**b**) α-cytidine, (**c**) β-cytidine, (**d**) 2′-deoxycytidI (α form) (**e**) 2′deoxycytidine (β form), (**f**) 3′-deoxycytidine, (**g**) 2′,3′-dideoxycytidine (Zalcitabine), (**h**) 5-aza-2′-deoxycytidine (Decitabine) and (**i**) cytosine. (The white color represents the contacts nearer to sum of the vdW radii, while red and blue color indicates shorter and longer contacts).

The 2D molecular fingerprints of cytosine, 2′-deoxycytidine (β form), and β-cytidine most closely resemble those of Ara-C. However, the visual comparison of the total 2D molecular fingerprints is difficult. The characteristic features of the local 2D fingerprint (2D FP) *d*_e_/*d*_i_ plots limited to O···H/H···O, C···H/H···C, N···H/H···N and H···H contacts were analyzed in depth. The highest contribution to 2D fingerprint is brought by weak H···H interactions, which are reflected by the cloud of scattered points with spikes at *d*_e_ + *d*_i_~2.0–2.3 Å, Figure 9. In Ara-C, these interactions are clearly stronger than in β-cytidine.

**Figure 10:**
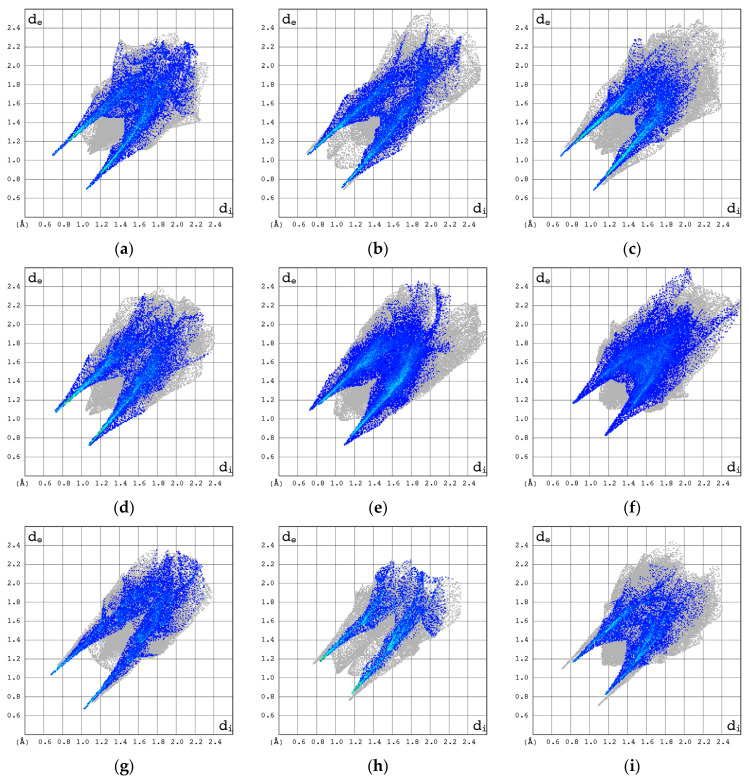
The 2D molecular fingerprints limited to OH interactions (**a**) Ara-C, (**b**) α-cytidine, (**c**) β-cytidine, (**d**) 2′deoxItidine (α form) (**e**) 2′deoxycytidine (β form), (**f**) 3′-deoxycytidine, (**g**) 2′,3′-dideoxycytidine (Zalcitabine), (**h**) 5-aza-2′-deoxycytidine (Decitabine) and (**i**) cytosine. (The white color represents the contacts nearer to sum of the vdW radii, while red and blue color indicates shorter and longer contacts).

In the 2D FP plots, Figure 10, the O···H/H···O contacts describing intramolecular O–H···O and intermolecular O–H···O and N–H···O hydrogen bonds are represented by symmetric spikes (“wings”). These sharp and small wings are located at *d*_e_ + *d*_i_~1.75–2.1 Å and cover a relatively small area of 22–31.5% of the total 3D HS. In β-cytidine, these interactions seem stronger than in Ara-C.

**Figure 11:**
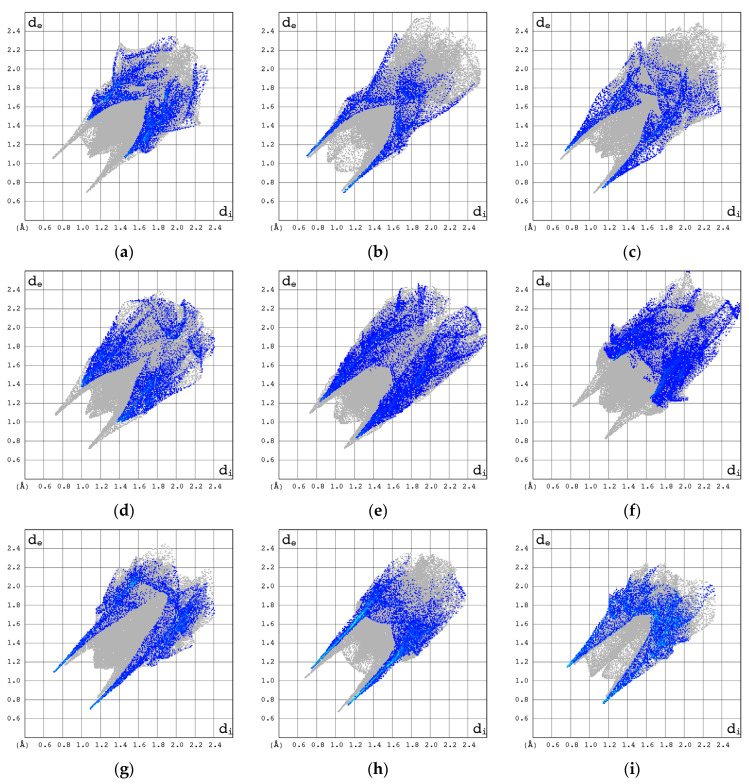
The 2D molecular fingerprints limited to NH interactions (**a**) Ara-C, (**b**) α-cytidine, (**c**) β-cytidine, (**d**) 2′-deoxycytidine (α form) (**e**) 2′deoxycytidine (β form), (**f**) 3′-deoxycytidine, (**g**) 2′,3′-dideoxycytidine (Zalcitabine), (**h**) 5-aza-2′-deoxycytidine (Decitabine) and (**i**) cytosine. (The white color represents the contacts nearer to sum of the vdW radii, while red and blue color indicates shorter and longer contacts).

The N···H/H···N contacts are represented by the external symmetric spikes sharp, and *d*_e_ + *d*_i_~1.8–2.9 Å and cover only about 12–23% of the total 3D HS, Figure 11. They are strong for Decitabine, suggesting the presence of NH···N interactions in this structure.

The ^14^N NQR parameters for the corresponding nitrogen atoms in Ara-C and cytidine differ significantly, suggesting a different binding mode in each crystal structure. The differences between the NH-limited 2D fingerprints of Ara-C and cytidine, Figure 10a–c, are significant and much greater than those between their HH- or OH-limited 2D fingerprints. Furthermore, according to the local 2D fingerprints the hydrogen bonds involving nitrogen are relatively weak in Ara-C when compared to α-cytidine, β-cytidine 2′-deoxycytidine β, Zalcitabine or cytosine.

**Figure 12:**
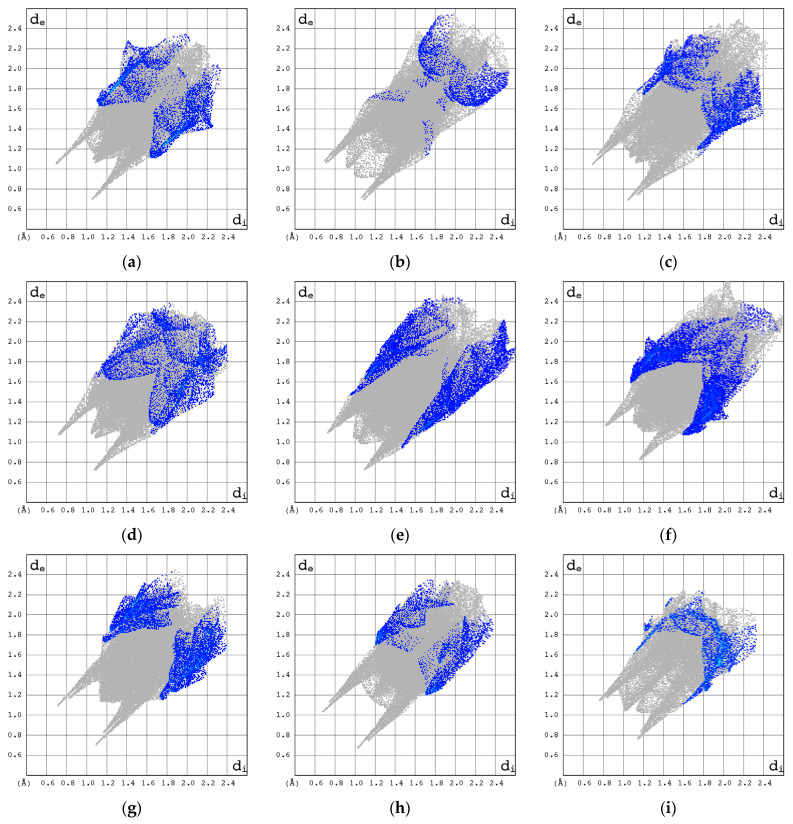
The 2D molecular fingerprints limited to CH interactions: (**a**) Ara-C, (**b**) α-cytidine, (**c**) β-cytidine,I), (**d**) 2′-deoxycytidine (α form) (**e**) 2′-deoxycytidine (β form), (**f**) 3′-deoxycytidine, (**g**) 2′,3′-dideoxycytidine (Zalcitabine), (**h**) 5-aza-2′-deoxycytidine (Decitabine) and (**i**) cytosine. (The white color represents the contacts nearer to sum of the vdW radii, while red and blue color indicates shorter and longer contacts).

The C···H/H···C contacts are of minor importance. They are represented by the most external wide spikes at *d*_e_ + *d*_i_~2.7–3.0 Å and cover 3.3–16.0% of the total 3D HS, Figure 12.

The impact of other contributions is negligible. The C···O/O···C contacts provide a tiny contribution of about 4.1% and they are represented by sharp wings, *d*_e_ + *d*_i_~3.40 Å, placed in the middle area of the entire fingerprint. The O···N and C···N/N···C, contacts bring negligible contribution of 1.2 and 1.0%, respectively. The contribution brought by C···N/N···C and C···C contacts, comes mainly from interlayer π···π stacking interactions.

Thus, the percentage of the three-dimensional Hirshfeld surface or enrichment ratios seems to indicate only general trends in intermolecular contacts.

#### 2.2.2. Strength of the Interactions

The differences in percentages in the 3D Hirshfeld surface between Ara-C and the nucleotides (2′-deoxycytidine, 3′-deoxycytidine or 2′,3′-dideoxycytidine) are relatively small compared to those between Ara-C and cytosine. The 3D HS surface, with its normalized contact distance d_norm_, shape index and curvature mapped over it, provides additional information about the nature of the non-covalent interactions in Ara-C, Figure 9. They help identify OH···O, NH···O and NH···N bonds involving donors and acceptors of the ligands. The energy of these interactions was calculated using a pair model, Table 7.

The intramolecular O–H···O hydrogen bond in sugar moiety of Ara-C linking OH at 2′ position of the sugar and OH group of CH_2_OH moiety is very short (R_O···O_ = 2.650 Å) and nonlinear (<OHO = 155.45°) occurs only in Ara-C. Intense red areas in the 3D HS near O and H from –OH, represent very low values of d_norm_ = −0.6830 a.u., shape index = −0.9965, and curvedness = −1.3652 a.u., which confirms existence of this strong bond. It stiffens structure and modifies the electron density within the glycone moiety, and as a consequence, the entire molecule. Therefore, it affects the ability of other atoms of the ligand to form hydrogen bonds.

The intermolecular hydrogen bonds are shown in the 3D HS surface by the areas that range in color from deep red to white. Deep red areas in the 3D HS localized near two H atoms (from –NH_2_) represent low values of d_norm_ = −0.4106 and −0.3885 a.u., shape index = −0.9561 and −0.9304 a.u., and curvedness = −2.0440 and −1.8137 a.u. They confirm the participation of the amine group, NH_2,_ in strong N–H···O hydrogen bonds, which are short (R_O···N_ = 3.048 and 2.983 Å) and nonlinear (<NHO = 163.7 and 145.73°). The –N= participates in weak, nearly linear, CH···N hydrogen bond of 3.562 Å. The small light red area near =N- and H from Ry sugar represent the values of d_norm_ = −0.8983 a.u. (shape index = 0.9512 a.u. and curvedness = −2.2851 a.u.). The –NRy participates exclusively in covalent bonds. The remaining red areas on 3D HS surface reveal OH···O intermolecular hydrogen bonds, which are short (R_O···O_ = 3.003 and 2.720 Å) and non-linear (<OHO = 159.64 and 172.71°).

The overall interaction energy partitioning suggests that the crystalline packing in Ara-C is primarily governed by electrostatic and repulsive strong N–H···O, O–H···O and C–H···N hydrogen bonds, followed by weaker N–H···O and C–H···O, which are mainly dispersive and repulsive (Table 7). In β-Cytidine and 2′-deoxycytidine strong N–H···O, O–H···N and O–H···O hydrogen bonds are electrostatic and repulsive.

Intramolecular OH∙∙∙O bonds closing the 5-membered ring occur only in Ara-C, while very weak CH∙∙∙O (O from =O) bonds closing the non-planar ring occur in all structures except cytosine. The –OH group in Ara-C is oriented toward the cytosine moiety, prompting it to engage in intramolecular hydrogen bond and form an additional heterocyclic quasi-ring, stiffening the entire structure. Therefore, the sugar component is crucial for the formation of the specific interaction pattern in the Ara-C crystal structure. The arrangement of hydroxyl groups, as well as their number, play a key role. All 2D molecular fingerprints indicate that the strongest interactions in each structure involve O and H contacts, specifically OH∙∙∙O and NH∙∙∙O hydrogen bonds. It is reasonable to assume that they will also have a significant impact on protein–ligand interactions.

#### 2.2.3. Differences in Binding Modes in the Solid State

The root-mean-square deviation of the binding modes (RMSD_BM), which shows the difference between the interactions in the solid-state structure of the Ara-C and studied ligand, is listed in Table 8.

The total RMSD_BM, which can be interpreted as binding distance, suggests that the binding mode in Ara-C is the most similar to that in 2′-deoxycytidine α, Decitabine and Zalcitabine. A comparison of the RMSD of the interactions in cytidine α and 2′-deoxycytidine β suggests that they are significantly different from the interactions in Ara-C, which is surprising. Despite the poor quality of the 3′-deoxycytidine structure, the RMSD_BM values do not differ significantly from the others.

However, the RMSD_BM of HH contacts in Ara-C is close to that in Decitabine, 2′-deoxycytidine, α and β and Zalcitabine. In contrast, the RMSD of OH and NH contacts in Ara-C are similar to those in cytidine β and Decitabine. In turn, the RMSD_BM describing CH contacts in Ara-C is similar to those in Decitabine, cytidine β, Zalcitabine and cytosine. Thus, some contacts are strengthened, others weakened upon inversion or deoxygenation. While this is obvious in relation to glycone, it also applies to aglycone moiety, in particular nitrogen contacts, which would seem to be unaffected by the inversion. This confirms the conclusions drawn from experimental research—the effect of a small structural change in glycone is transferred through the system of bindings to distant sites in aglycone. It is reasonable to assume that such small changes will also have a significant impact on protein-ligand binding modes.

### 2.3. Binding to Deoxycytidine Kinase (dCK)

#### 2.3.1. Molecular Docking Results

Human deoxycytidine kinase (dCK) is a well-known enzyme encoded by the human DCK gene, a potential suicide gene. It catalyzes the first step in the nucleoside salvage pathway, converting natural deoxyribonucleosides to their monophosphate forms [3]. dCK also plays a key role in the phosphorylation of numerous nucleoside analog prodrugs routinely used in anti-cancer and anti-viral treatments. The rate-limiting step in the activation of Ara-C is deoxycytidine kinase-dependent phosphorylation.

The crystal structure of dCK in complex with Ara-C, 1P5Z [49] was obtained from the PDB database. Three of the dCK residues (Tyr86, Asp133 and Glu197) involved in the protein-ligand binding are in close proximity to the native ligand (2 Å), fourteen (Phe137, Gln97, Glu53, Phe96, Arg128, Trp58, Ile30, Tyr86, Ile200, Leu82, Val55, Arg104, Met85 and Lys34) are in further proximity of 3 Å and three remaining residues (Arg194, Leu141, and Ala100) in slightly distant proximity of 4 Å. Prior to docking, the native ligand that co-crystallized with the dCK was removed from the structure. The root-mean-square deviation (RMSD) of the best pose relative to the actual ligand redocked in its own binding site was only 0.113 Å. Thus, the quality of the docking process is very high. The same protocol was used to dock other ligands into the rigid dCK structure. In order to compare the docking quality of 2′-deoxycytidine and Gemcitabine, a re-docking task was also performed. They were docked in the original active sites derived from structures 1P60, 1P61 and 1P62 [49] (the RMSD of the best poses did not exceed 0.5 A). The weak point of classical molecular docking is the lack of flexibility of the residues. Therefore, we verified the results of the docking using two different techniques, the flexible ligands and the flexible residues, the latter being applied to the extent possible. The variations in binding modes are found to be meaningless (within the limits of repeatability of results). Molecular dynamics simulations performed using a coarse-grained approach, confirmed the conclusions drawn from flexible molecular docking. Under crystalline conditions (native state) only a few side chains: Asn77, Gly78, Thr64, Glu120, Ser63 and Gln79 show increased flexibility. However, the root-mean-square-fluctuation (RMSF) of a structure, which is the time average of the root mean square deviation (RMSD) of atomic structures did not exceed 1.54 Å. Assuming very high side-chain mobility (completely free small protein chains) Asn77, Leu221, Lys222, His218 and Gly78 have been detected as more flexible, with the RMSF value not exceeding 3.5 Å. The radius of gyration of approximately 16.5 nm changed relatively little, by 1.6 and 3.1%, respectively. It is worth noting that the binding site is stable even when the pocket is empty. The variations in binding modes were found insignificant and irrelevant from the point of view of our research, falling within the range produced by the various docking techniques and do not affect the final conclusions.

**Figure 13:**
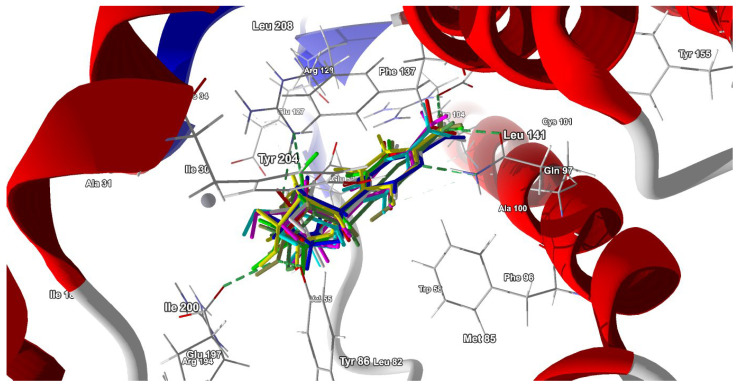
The best docking poses of the ligand in complexes with dCK protein. The protein backbone is represented as a cartoon, the binding cavity residues are shown as thin sticks and the docked ligands are shown as color sticks (Ara-C in green, 2′-deoxycytidine in yellow, cytidine in cyan, 5-azacytidine in magenta, 3′-deoxycytidine in white, Zalcitabine in blue, Decitabine in dark green and 3′-deoxy,3′,4′-didehydrocytidine in red). The hydrogen bonds linking Ara-C to the dCK residues are shown using dashed green lines.

The docking results are presented in Table 9. The best docking poses are shown in Figure 13.

The total energy is the highest for Ara-C, while the protein-ligand term is the highest for Gemcitabine. Hydrogen bonds and steric interactions (including hydrophobic ones) are the strongest in Ara-C, followed by Gemcitabine, 2′-deoxycytidine and Decitabine.

#### 2.3.2. dCK-ligand Binding Modes

**Figure 14:**
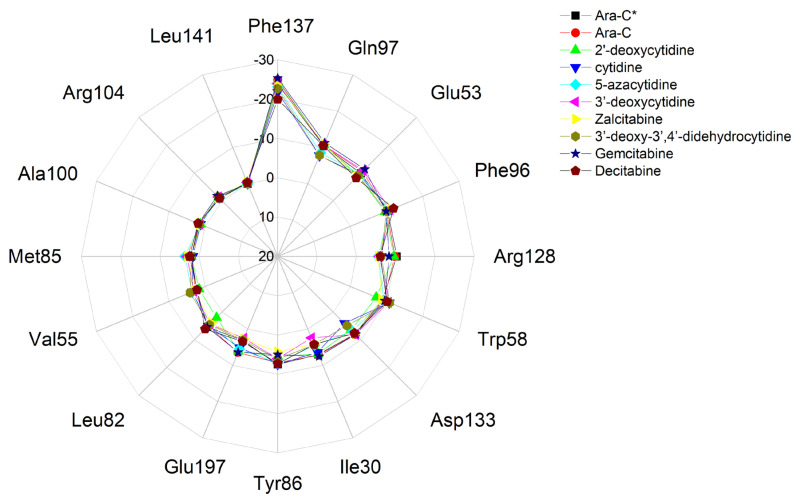
The radar plot visualizing the protein-ligand binding mode (Ara-C*—native ligand).

The interaction of the ligands with dCK involves 15 to 18 residues in total (the 14 residues are constant), Table 10. A radar plot demonstrating the differences in binding modes is shown in Figure 14.

Among the ligands, 2′-deoxycytidine and cytidine bind to the largest number of the residues, while 3′-dehydro-3′,4′-dideoxycytidine binds to the smallest. The differences in the binding modes between the real and the redocked ligands are negligible. The largest variation in binding strength is observed for Gln97, Arg128, Asp133 and Ile30. It is worth noting that three residues in the nearest vicinity (Tyr86, Asp133 and Glu197) are not most strongly bound in the complex. The modes of binding of the individual ligands to the protein treated as a whole can be compared in a quantitative manner using, as proposed by us, parameter root-mean-square deviation of binding mode (RMSD_BM), Table 11.

As follows from Table 12, Decitabine, Gemcitabine and 2′deoxycytidine are closest to Ara-C in terms of the complete binding mode (the corresponding RMSD_BM values are the smallest). The RMSD_BM*, RMSD_BM** and RMSD_BM*** describing the differences between the native and redocked ligands or two native ligands are small.

**Figure 15:**
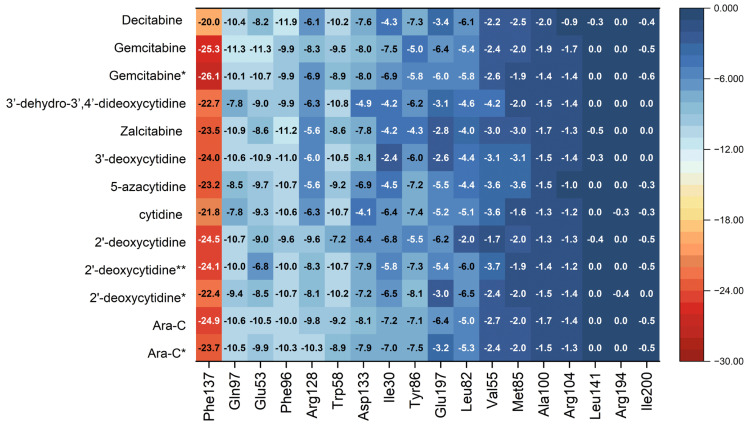
The binding strength of the ligands to individual residues (Ara-C* 1P5Z, 2-deoxycytidine* P160, 2-deoxycytidine** P161, Gemcitabine* P162 are listed as reference). The heat map visualizes the binding mode in red-yellow-blue scheme, with dark red indicating strong interactions and dark blue indicating very weak ones.

The heatmap shown in Figure 15 visualizes the binding mode in detail and reveals the importance of the individual binding components.

The heat map of binding strength for each ligand versus residue allows for the identification of the strongest and most stable components of the binding mode. It shows that the binding between Phe137 residue and ligand (Ara-C and its analogues) composed of π-π stacking and hydrophobic interactions is the strongest. Moreover, this strong binding is supported by a much weaker interaction between the ligand and Phe96. It is worth noticing that Phe often acts as a “steric gate” preventing the incorporation of nucleoside triphosphate due to steric hindrance with 2′-OH [50]. Because the 2′-OH of the glycone in Ara-C points in an opposite direction, it does not generate any steric clashes in dCK.

The ligands that are deprived of the -OH group at the 2′ position are Ara-C, 2′-deoxycytidine, Zalcitabine, Decitabine and Gemcitabine. Surprisingly, with Decitabine as a ligand, the binding to Phe137 is strongly weakened. Therefore, additional nitrogen has a strong modulating effect on the density distribution: instead of NH···O, weaker NH···N hydrogen bonds are formed.

In general, the ligands’ ordering according to the decreasing binding strength is as follows:

Phe137 > Gln97 > Glu53 > Phe96 > Arg128 > Trp58 > Asp133 > Ile30 > Tyr86 > Glu197.

The remaining ligands Leu82, Val55, Met85, Ala100, Arg104, Leu141, Arg194 and Ile200 are insensitive to ligand type and play a marginal role in the binding.

**Figure 16:**
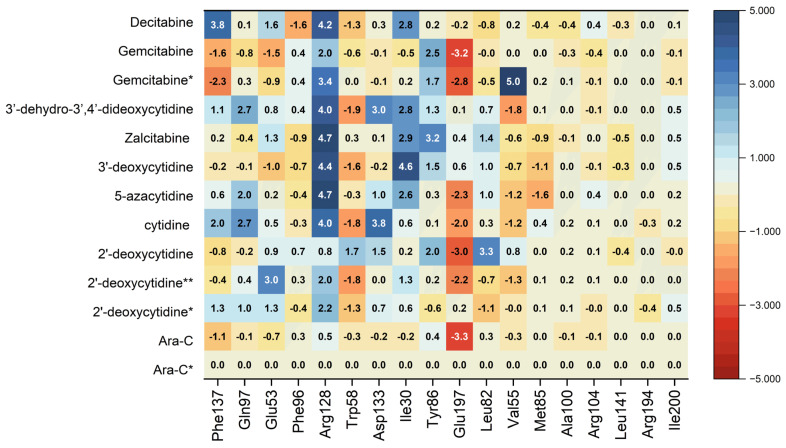
Difference heat map revealing the differences in the binding strength of ligands to individual residues (Ara-C* 1P5Z, 2-deoxycytidine* P160, 2-deoxycytidine** P161, Gemcitabine* P162 are listed as reference). Thered-yellow-blue scheme, where red indicates bond strengthening and blue indicates bond weakening was used.

The differences in ligand binding strength to individual residues can be easily compared and analyzed by means of the difference heat map, Figure 16.

The map shown in Figure 16 shows the deviations of the binding energy with respect to the native ligand treated as a reference.

As follows from Figure 16, the greatest differences between the ligands occur in their binding to the residues: Arg 128, Ile30, Glu197 and Asp133. This finding is in line with Sabini et al. [49] suggestion that the interaction between Arg128 and the hydrogen-bond acceptor at the sugar 2′-arabinosyl position of Ara-C is relevant for the biological activity. The binding of Arg128 to ligands is strongly weakened for 5-azacytidine, 3′-deoxycytidine, Zalcitabine, 3′-dehydrio-3′,4′-dideoxycytidine and Decitabine and strong only in Ara-C, Gemcitabine and 2′-deoxycytidine. Moreover, the binding to Ile30 is strongly weakened nearly for the same set of ligands: cytidine, 5-azacytidine, 3′-deocycytidine, Zalcitabine, 3′-dehydrio-3′,4′-dideoxycytidine and Decitabine. It is also the strongest in Ara-C, Gemcitabine and 2′-deoxycytidine. The binding to Glu197 is strongly enhanced for 2′deoxycytidine, cytidine, 5-azacytidine and Gemcitabine. Therefore, the whole picture is more complicated, and our analysis reveals many more important components of protein-ligand bonds than just the binding of the ligand to Arg128.

**Figure 17:**
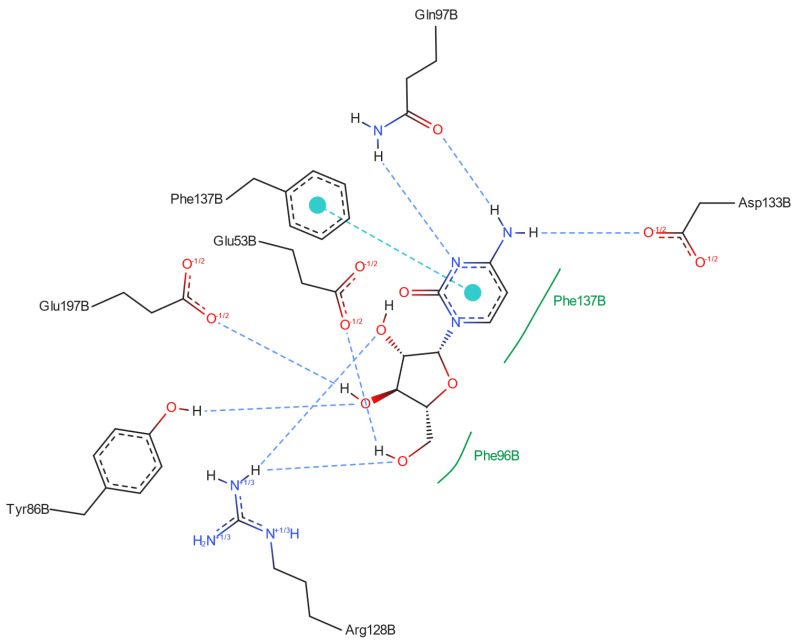
The binding mode in Ara-C–dCK complex (hydrogen bonds are depicted in blue, hydrophoblic contacts in green and π···π interactions in cyan).

Identifying the atoms involved in the hydrogen bonds connecting ligands to residues can shed some light on the moieties in the ligands that are most important for effective binding, Figure 17 and Table 12.

As follows from Table 12 and Figure 17, NH_2_ and –N= play a key role, as they bind Gln97 residue using strong NH···O and NH···N hydrogen bonds and Asp133 using weaker NH···O bonds. In addition, OH from CH_2_OH moiety is highly important, binding to Glu53 through OH···O and Arg128 through NH···O. However, only 2′deoxycytidine, Gemcitabine and Decitabine bind to Arg128 via hydrogen bonds. Moreover, protein-ligand binding is strongly modulated by multiple OH···O hydrogen bonds connecting the glycone hydroxyl groups to Tyr86, Glu197 and Arg128 (only in Ara-C). Therefore, the lack of a hydroxyl group at the 2′ position has a similar effect on binding as its inversion or replacement with fluorine. However, dihydroxylation at the 2′ and 3′ positions of the glycone has no significant effect on the strongly binding components.

An important factor from the point of view of binding is the conformation of the glycone moiety. The angle describing the conformations of the ligands differs by at most about 30 degrees. However, the orientation of the glycone varies significantly for the different polymorphic forms, e.g., cytidine and 2′-deoxycytidine, Table 13. In terms of conformation, Zalcitabine, β-cytidine, and Decitabine are most similar to Ara-C.

The conformation of the glycone in Decitabine, 2′-deoxycytidine and Ara-C after docking in the active pocket is almost the same as in the solid state. For the remaining ligands, the differences are quite significant. The OH group at position 2′ of the glycone is an important factor in conformational stability. It stiffens the structure by forming a short but bent OH···O hydrogen bond with the –OH group of CH_2_OH moiety. This bond is unique, occurs only in Ara-C and strongly modifies the electron density distribution in glycone, and consequently, in the entire molecule.

Heat maps, RMSD_BM distances and analysis of the ligand atom contribution to the hydrogen bonds show that the binding modes of Gemcitabine, Decitabine and 2′-deoxycytidine to dCK are closest to the binding of Ara-C to dCK. Moreover, the heat maps help identify the main components of the binding in solid and protein–ligand complex, which are nitrogen –NH_2_, –N(3)= and –OH. However, predicting the activity from the results of a docking study is time-consuming.

### 2.4. Relative Reactivity of the Ligands

We recently noticed a relationship between relative reactivity and relative ligand-protein binding strength that allows effective screening of SARS-RdRp protease ligands [51]. We decided to check whether the recently developed relative reactivity parameters would also prove useful for Ara-C and their analogues binding to deoxycytidine kinase, dCK. The frontier molecular orbitals (FMO) theory is well-known for conceptualizing chemical bonding and reactivity in terms of interactions between orbitals located at the boundary between occupied and unoccupied. Lowest unoccupied molecular orbital (LUMO) accepts electrons, and its energy corresponds to an electron affinity (EA). The highest occupied molecular orbital (HOMO) donates electrons, and its energy is related to ionization potential (IP). Low IP and high EA are associated with high nucleophilic and electrophilic properties, respectively. Thus, the HOMO–LUMO gap is an important measure for the determination of the charge transfer within molecule. Theoretical global indices (absolute electronegativity, χ; absolute hardness, η; electrophilicity index (reactivity), ω; softness, S; electro-donating power, ω-; electro-accepting power ω+; net electrophilicity, Δω; and a maximum number of electrons transferred in a chemical reaction, ΔN_max_) provide additional information on the ligand’s reactivity. Table 14 shows the HOMO and LUMO energies, the HOMO–LUMO gap and global reactivity indices for ligands evaluated at the M062X/6-311G(d,p) level of the theory.

The ligands can be arranged using the decreasing HOMO–LUMO gap as follows:

Decitabine > 5-azacytidine > Ara-C > Zalcitabine > 3′-deoxycytidine >2′-deoxycytidine > Gemcitabine

Three compounds, Decitabine, 5-azacytidine and Ara-C, which have a higher HOMO–LUMO energy gap, are more stable than the other ligands. Furthermore, their very high value of the absolute hardness but small value of softness suggests a high degree of stability.

The χ parameter describes the tendency to donate/accept electrons, while η measures the ease with which this process occurs. Both are high for 5-azacytidine, and low for Ara-C and Decitabine. The electrophilic power, which measures the capacity of an electrophile to accept the maximal number of electrons in a neighboring reservoir of electron pool, is represented by the descriptor ω (the global electrophilicity index) and falls within the range of 1.683–2.498 eV, Table 14 The reactivity of the ligand measured using ω, reveals the following trend: cytidine > 5-azacytidine > 3′-deoxycytidine > Decitabine > Zalcitabine > 2′-deoxycytdine > Gemcitabine > Ara-C.

Thus, the change in sugar moiety leads to a decrease in electrophilic activation from 2.568 to 1.683 (1.768 for native ligand), while additional nitrogen at –N(5) site leads to its decrease to 1.859. The system’s tendency to acquire electrons from the environment (evaluated using reactivity) is observed to be very high for Ara-C and Gemcitabine, while very low for cytidine. Thus, Ara-C and Gemcitabine have relatively low substrate selectivity, which means they can inhibit a wide range of proteins. Ara-C has the lowest local electron-donating power, ω^+^, electron-accepting power, ω^−^, and overall electrophilicity, Δω. A larger ω^+^ value for Ara-C than 2′-deoxycytidine corresponds to its superior ability to accept charge, but small ω^−^ value for Ara-C enhances the ligand’s ability to donate electrons. However, cytidine and 5-azacytidine possess an unusually low-lying LUMO level, indicating their susceptibility to molecular reactions with nucleophiles. The low-lying HOMO level for cytidine, Decitabine and 5-azacytidine suggests they are easier than Ara-C in participation in molecular reactions with electrophiles. Two new reactivity descriptors, the so-called relative electron-donating power, R^+^, and the relative electron-accepting power, R^−^, were defined recently by us [51]. They describe the relative ability of the ligand to accept and donate charges, respectively.

The three ligands Ara-C, Gemcitabine and natural 2′-deoxycytidine are involved in strong hydrogen bonds with dCK residues, while Zalcitabine and 3′-deoxycytidine (located on the border of this area), binds to dCK much weaker. The relative R^+^ and R^−^ with Ara-C as reference show that Gemcitabine, Zalcitabine and Decitabine are closest to it in terms of electron donating/electron withdrawing properties. On the other hand Ara-C, Gemcitabine and Decitabine are closest to 2′-deoxycytidine (treated as reference), while 5-azacytidine and 3′-deoxycytidine are closest to cytidine (treated as reference).

### 2.5. Quantitative Structure–Property Relationships (Binding Affinity, Relative Reactivity and Biological Activity)

**Figure 18:**
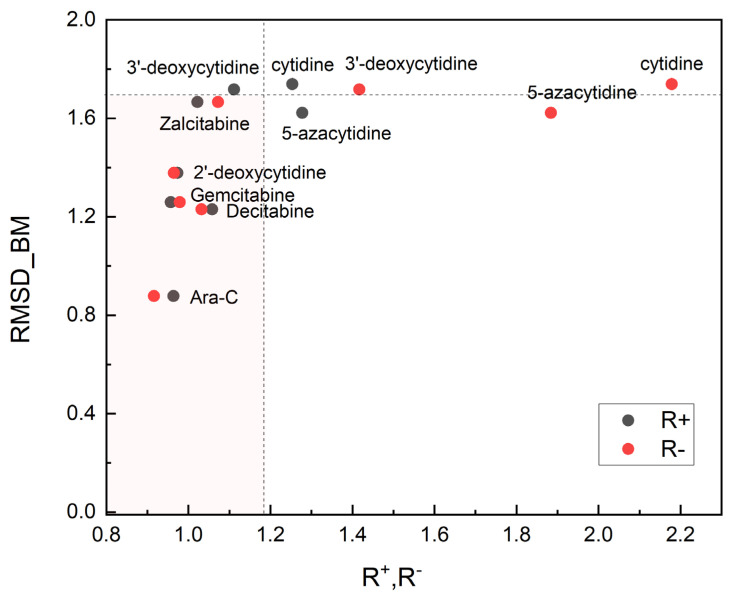
The correlation between relative binding mode (RMSD_BM) and the relative reactivity R^+^ and R^−^.

The root-mean-square deviation of the binding modes (RMSD_BM) is non-linearly correlated with the relative reactivity power R^+^ and R^−^, Figure 18 and shows a distinct separation between the studied compounds.

**Figure 19:**
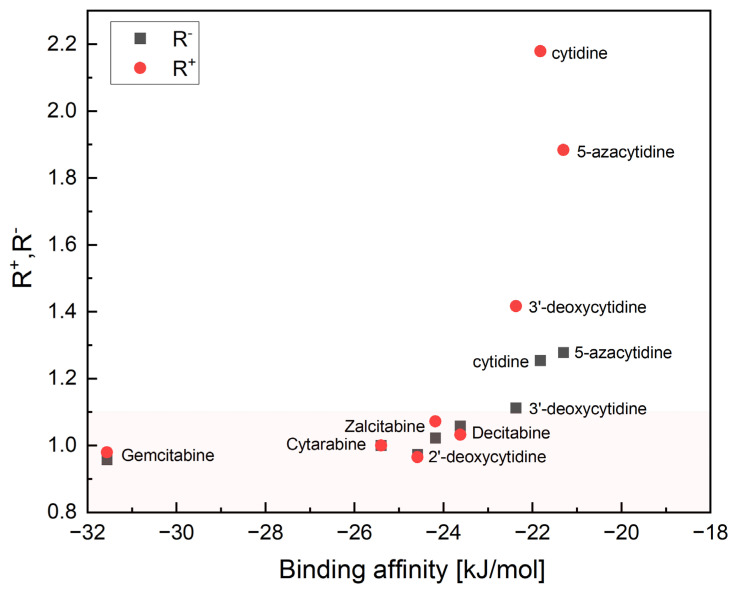
The correlation between relative binding affinity and the relative reactivity R+ and R−.

Furthermore, the binding affinity is also non-linearly correlated with the relative reactivity R^+^ and R^−^, Figure 19.

As can be seen from Figure 18 and Figure 19, a small structural change, the inversion of the hydroxyl group at the 2′ position of the sugar or removal/replacement of glycone substituents, leads to a significant change in relative reactivity, binding mode and binding affinity. The root-mean-square deviation of the binding modes (RMSD_BM), binding affinity and R^+^ and R^−^ can be treated as combined screening parameters: smaller R values means stronger binding, smaller RMSD_BM and binding affinity means increased anti-leukemic efficiency. Anti-viral ligands (3′-deoxycytidine, Zalcitabine) are blocked at the 3′-hydroxyl group of DNA, which prevents elongation of nascent DNA. The RMSD_BM, R^+^ and R^−^ for them are higher than for anti-leukemic Ara-C, Gemcitabine and Decitabine, Figure 18. The light pink area in Figure 18 indicates ligands with the low and close values of the relative R^+^ and R^−^ parameters, which bind easily to dCK: Ara-C, Gemcitabine, Decitabine (RMSD_BM < 1.4; anti-cancer) and Zalcitabine (RMSD_BM > 1.4; anti-viral). The R^+^, R^−^ values higher for Gemcitabine than Ara-C suggests that Gemcitabine should be slightly more effective than Ara-C. Indeed, Gemcitabine has broad anti-leukemic activity across different AML subtypes and is more effective than Ara-C, both in vitro and in vivo [52]. Our results are also in a good agreement with the preclinical studies, which indicated that Decitabine is a more effective anti-leukemic agent than 5-azacytidine [53], but five times less effective than Ara-C [54]. The mechanism of action of 5-azacytidine is different and involves primarily DNA hypomethylation. 5-azacytidine and Decitabine are used in combination with Ara-C [55] due to synergistic induction of apoptosis. Moreover, the IC50 determined against HL-60 cell lines for 5-azacytidine or Decitabine is significantly higher than that of Gemcitabine, indicating that these two compounds are less effective than Gemcitabine [56]. On the other hand, cytidine does not have any anti-cancer properties, which is supported by distinct R^+^ and R^−^ and high RMSD_BM. The binding affinity, which is the highest for three ligands: Gemcitabine, Ara-C, and natural 2′-deoxycytidine and the lowest for cytidine reflects RMSD_BM well.

Comparing the relative reactivity, binding mode, or binding affinity of ligands with their biological activity is generally challenging due to variations in the scope of action, Table 15, as well as differences in protocols, doses, methods of administration, and timing of measurements used in different cell-line studies. Furthermore, therapeutic efficacy is limited by other factors including the rapid elimination of intracellular CTP forms in target cells.

Despite the different spectrum of activity, all ligands share a common feature-require metabolic activation via phosphorylation catalyzed by dCK. Thus, low levels of dCK enzyme activity [57], mutations in dCK [58] or increased levels of active triphosphate metabolites [59] contribute to variability in their response. dCK has recently been recognized as a promising new target for BRCA2-deficient cancers (an alternative to PARP inhibitors) [59], an agent to reduce the symptoms of encephalitis (inflammation of the brain) [60] and the cause of Gemcitabine resistance in pancreatic cancer patients (due to dCK inactivation) [61]. Furthermore, dCK deficiency or weak binding to dCK are the rate-limiting factor for phosphorylation and in a consequence biological activity. The methods proposed for comparing ligand reactivity and protein-ligand binding modes show promise for screening ligands or searching for drug improvements.

## 3. Materials and Method

### 3.1. Material

The Ara-C samples were purchased from Sigma Aldrich, Poland and Slovenia and used without further purification. The purity of the powdered samples was confirmed to be not lower than 98%. The experimental study was performed using a 0.5 g sample of Ara-C. To avoid hydration, the sample was transferred to a sealed quartz test tube and used for NMR relaxation measurements without exposing the sample to the air.

### 3.2. Methods

#### 3.2.1. Nuclear Quadrupole Resonance (NQR)

The ^14^N NQR frequencies in Ara-C cover the range below 3 MHz, for three different types of nitrogen including –N=, which presents an experimental challenge. Furthermore, the direct measurement of NQR on a small sample (approx. 0.5 g) is practically not possible due to the very weak signal (low signal-to-noise ratio).

Therefore, an indirect measurement technique is used to determine the NQR frequency, which involves measuring the proton NMR signal using double nuclear quadrupole double resonance techniques. In challenging cases, such as Ara-C, a multi-step procedure is necessary.

*In the first step*, the ^1^H-^14^N cross-relaxation (CR) [62] spectrum was measured using fast field cycling (FFC) relaxometry. The standard CR experiment uses three consecutive time intervals with different values of the static magnetic field:

(i) Polarization field $B_{P}$ for time ${\;t}_{P}\geq 3T_{1H}\left(B_{P}\right)$, where $T_{1H}\left(B_{P}\right)$ is the proton spin-lattice relaxation time in the field $B_{P}$ (an equilibrium proton magnetization condition).
(ii) Relaxation (also called “mixing”) field $B_{R}$ for time τ, where the proton magnetization relaxes towards the equilibrium value with a time constant $T_{1H}\left(B_{R}\right)$.
(iii) Acquisition field $B_{A}$, at which the proton NMR signal amplitude, which is proportional to the proton magnetization at the end of $B_{R}$ interval, is measured.

The experiment is repeated with a different relaxation field $B_{R}$ and relaxation time τ to determine the full dispersion of spin-lattice relaxation time $T_{1H}\left(B_{R}\right)$. When the proton NMR Larmor frequency $\nu_{H}$ matches one of the ^14^N NQR frequencies ${\;\nu }_{Q}$ or $\nu_{H}=\frac{\gamma_{H}}{2\pi }B_{R}=\nu_{Q}$, a drop in $T_{1H}\left(B_{R}\right)$ is frequently observed. This phenomenon is commonly known as “quadrupole dips” [62] and is frequently used to indirectly determine the NQR frequencies.

In the case of Ara-C, as for favipiravir [63], the $T_{1H}\left(B_{P}\right)$ significantly exceeds typical values. Such conditions are unfavorable from the experimental point of view because the sample cannot be properly polarised and measurements are very time-consuming. A modification of the classical technique in which the polarization period ($B_{P}=0$) is removed from the experiment and the proton magnetization at the beginning of the $B_{R}$ interval is zero is an effective solution. The magnetization of the proton increases towards the equilibrium value according to the formula $M=M_{0}(1-e^{-\frac{\tau }{T_{1H}\left(B_{R}\right)}})$. For $B_{R}$ values at which the ^1^H proton NMR transition frequency $\nu_{H}$ matches one of the ^14^N NQR transition frequencies $\nu_{Q}$, the proton system is additionally polarised by the transfer of polarisation from nitrogen NQR levels to proton NMR levels with a characteristic cross-relaxation time $T_{CR}$. The additional “quadrupole peaks” appear in the CR spectrum when the condition $T_{CR}<T_{1H}\left(B_{R}\right)$ is met.

The ^1^H-^14^N cross-relaxation spectrum of Ara-C was measured by this modified cross-relaxation technique. Although the peaks are well located in the spectrum, the resolution is relatively low due to line broadening due to the Zeeman effect as well as the Zeeman shift with respect to the pure NQR frequencies in the zero field [64].

*In the second step*, the techniques of multiple frequency sweeps and two-frequency irradiation [25,26] were used. They enable a more precise determination of the frequencies of the NQR triplets ν_+_, ν_−_, and ν_0_ at the three nitrogen positions in an Ara-C molecule. In the search for the ^14^N NQR frequencies at the pyrazine nitrogen positions, we applied multiple frequency sweeps of the *rf* magnetic field in the frequency range 2.8–4 MHz and performed the ν_H_ scan.

#### 3.2.2. Computational Modeling-Density Functional Theory

##### Single Molecules and Clusters

The calculations for the single molecules and clusters were carried out using Gaussian 16 rev. C01 [65]. The quantum chemical calculations required for the QTAIM analysis were carried out within the density functional theory (DFT) approach rooted in the Kohn–Sham [66] theorem, generalized by Levy [67]. The hybrid meta exchange-correlation functional, with a double amount of non-local exchange, M062X [68] and an all electron split-valence basis set 6-311+G(d,p) was used. As we have shown previously, M062X provides a reliable electron density distribution in single molecules and cluster systems with non-covalent interactions [68]. Providing useful accuracy and favorable algorithmic complexity, this approach is an attractive compromise. The positions of the hydrogen atoms have been optimized. X-ray crystallography typically cannot directly determine the positions of light atoms at standard resolutions. The full description of the clustering technique can be found in our previous papers [63,69,70].

The theoretical reactivity indices defined based on the frozen core approximation (the Koopmans theorem [71] by Par and Pearson [72], Gázquez [73], Chattaraj [74] and Latosińska [51]: the absolute electronegativity [χ = −(E_LUMO_ + E_HOMO_)/2; eV]; absolute hardness [η = (E_LUMO_ − E_HOMO_)/2; eV)]; electrophilicity index (reactivity) [ω = χ^2^/2η; eV]; softness [S = 1/η; 1/eV]; electro-donating power [ω-; eV]; electro-accepting power [ω+; eV]; net electrophilicity [Δω = ω^+^ + ω^−^; eV]; maximum number of electrons transferred in a chemical reaction [ΔN_max_]; relative electro-donating power, $\left[R^{+}=\frac{\omega_{ligand}^{+}}{\omega_{reference\;ligand}^{+}}\right]$ and relative electro-accepting power: ${[R}^{-}=\frac{\omega_{ligand}^{-}}{\omega_{reference\;ligand}^{-}}]$ were evaluated at M062X/6-311G(d,p).

##### Solid-State

All solid-state quantum chemical calculations were carried out within the CASTEP [75] code. Different nonlocal generalized gradient approximation (GGA) functionals depending on both ρ and dρ/dr, namely RPBE (revised Perdew, Burke, and Ernzerhof) [76], PBE (Perdew, Burke, and Ernzerhof) [77] with the Tkatchenko–Scheffler (DFT-TS) correction for dispersion [78] was probed. The use of the gauge including projector-augmented waves (GI)PAW [79] exploiting the full translational symmetry of a crystal and on-the-fly generation ultrasoft (OTFG) potentials provided an excellent balance of speed and accuracy. It has no basis set superposition error and guarantees monotonic convergence to the target wavefunction. The sampling of the Brillouin zone was carried out with the Monkhorst and Pack [80] scheme (reciprocal space-based technique).

The components of the electric field gradient (EFG) tensor, a second-rank symmetrical electric field gradient (EFG) tensor, $q_{ii}=\frac{\partial^{2}V(r)}{\partial x_{i}^{2}}$ (*i* = *x*, *y* and *z*; *V*(*r*)—external electrostatic potential), satisfying the relationship $\left|q_{xx}\right|\leq \left|q_{yy}\right|\leq \left|q_{zz}\right|$, were calculated at the selected level of the theory. They were produced using the following tensor:

(1) $$\nabla E_{ij}=-\frac{1}{4\pi \varepsilon_{0}}\int_{-\infty }^{\infty }\left[\frac{3r_{i}r_{j}-\delta_{ij}{\left|r\right|}^{2}}{{\left|r\right|}^{5}}\right]\rho \left(r\right)dr$$

where (*r*) is the electron density, *r_i_* is the projection of the r vector onto *x*, *y*, and *z*-axes, and *δ_ij_* is Dirac’s delta function. The three eigenvalues (principal components are pairwise independent of each other) and three eigenvectors (describing the orientation of the principal axis with respect to an arbitrary frame) fully characterize the EFG tensor. Thus, EFG may be completely represented using only two parameters: the quadrupole coupling constant *e*^2^*qQ*/*h* = *e*^2^*q_Z_Q*/*h* and the asymmetry parameter *η*. Both are related to the ^14^N NQR frequencies ν_+_ and ν_−_ according to the following equations:

(2) $$\left|\frac{e^{2}Qq}{h}\right|=\frac{2}{3}\left(\nu_{+}+\nu_{-}\right)$$

(3) $$\eta =\frac{3\left(\nu_{+}-\nu_{-}\right)}{\nu_{+}+\nu_{-}}$$

On the other hand, the resonant frequencies of the ^14^N NQR are related to these parameters through a set of equations:

(4) $$\nu_{+}=\left|\frac{e^{2}Qq}{4h}\right|(3+\eta )$$

(5) $$\nu_{-}=\left|\frac{e^{2}Qq}{4h}\right|(3-\eta )$$

(6) $$\nu_{0}=\nu_{+}-\nu_{-}=\left|\frac{e^{2}Qq}{2h}\right|\eta$$

where a nuclear quadrupole moment for ^14^N equal to 2.044 fm^2^ is assumed [81].

#### 3.2.3. Hirshfeld Surfaces (3D HS)

The 3D Hirshfeld surfaces (3D HS) technique was used to investigate intermolecular interaction patterns in solids [17,78]. Three-dimensional HS is constructed using the function of the molecular weight (a quotient of the promolecule and the electron density of the procrystal). It describes the outside contour of a molecule’s space in a crystalline environment. The surfaces mapped across 3D HS descriptors d_norm_, shape index, and curvedness were analyzed [17]. The intermolecular interactions were visualised in the d_norm_ surface projected over 3D HS. The red-white-blue scheme was utilized, with red indicating short-range connections like hydrogen bonds, white representing contacts with van der Waals radii, and blue representing the remaining considerably longer interactions. The flatness of the surface was characterized by the shape index and curvedness of the 3D HS projected across it. The 3D HS was decomposed into a 2D molecular fingerprint (2D FP) map. This map plots the distances of each surface point to the neighboring interior and exterior atoms (d_i_ versus d_e_) [82] and describes the distribution of the molecule interactions with its environment. The enrichment ratio, E_XY_, a descriptor defined as the ratio of the proportion of actual connections in a crystal to the theoretical fraction of random interactions [83] was calculated based on 2D FP. It denotes a proclivity to make or avoid contacts.

#### 3.2.4. Molecular Docking

The molecular docking (MD) technique is used to model the interaction between a small molecule (ligand) and a protein (target) at the atomic level [84,85,86]. It is a crucial method for characterizing new synthesized compounds and predicting their activity [87,88,89,90] or characterizing protein-ligand binding modes [51]. However, knowledge of both the ligand structure and a reliable 3D crystallographic structure of the protein are required to obtain credible results. Three-dimensional (3D) molecular structures of the Ara-C, cytidine, 2′-deoxycytidine, 3′-deoxycytidine, 5-azacytidine, Zalcitabine and Gemcitabine have been optimized using Gaussian 16, rev. C01, at the M062X/6-31+G(d,p) level of theory. Structures of the target (1P5Z) and referenced complexes (1P60, 1P61 and 1P62) [49] were retrieved from the Protein databank PDB database (http://www.rcsb.org/pdb, accessed on 20 December 2023).

The conversion of the files with receptor and ligand structures to the .pdbqt format was made using MGLTools ver. 1.5.7. Molecular docking was performed using automated docking tools AutoDock ver. 4.2.6 [91] and AutoDock Vina ver. 1.2.3 [92]. Before docking the ligands, the native ligand that co-crystallized with dCK and water molecules was removed from the structure. The protonation state of the protein was checked and corrected prior to docking. To assess the docking process’s quality, a redocking task was performed. The redocking protocol was effective if the pose’s root-mean-square deviation (RMSD) from its conformation in the parental structure did not exceed 3 Å (the RMSD values for the studied ligands did not exceed 0.5 Å). The new ligand was docked into the rigid protein structure using two separate techniques: template docking and docking with defined search space around the active site. The grid box of size 9–15 Å was centered on the active site. After docking, the optimal poses that resulted in protein–ligand complex stabilization with the highest docking score were chosen and studied further. The molecular docking results were validated using a Genetic Evolutionary Method for molecular DOCKing (GEMDOCK) [93]. This technique also utilizes a genetic algorithm, but with a distinct evolution operator. The energy of the interactions was described using the sum of the piecewise linear potential (PLP) term (steric, van der Waals and hydrogen bonding interactions), and the Coulomb term (electrostatic interactions). Since the lack of protein flexibility is one of the greatest limitations in molecular docking, some flexibility of selected residues was assumed in GEMDOCK. The molecular dynamics simulations have been performed using coarse grain technique [94,95]. The total number of generated models was equal to 50,000, 2% of which were selected and 10 best used in the further comparative analysis. The binding affinity was estimated using the Gehlhaar model [96] with original parameterization and using PRODIGY [94,95]. The final 2D and 3D visualizations of the binding modes were made using PoseEdit [97] and VMD [98].

#### 3.2.5. Comparison of the Differences in the Binding Modes

##### Root-Mean-Square Deviation of the Binding Mode

The average deviation between the binding modes was calculated using the newly defined quantity: root-mean-square deviation of the binding modes (*RMSD_BM*). It was calculated as follows:

(7) $$RMSD\_BM(P,Q)=\sqrt{\frac{1}{n}\sum_{i}{\left(p_{i}-q_{i}\right)}^{2}}$$

where *p_i_* and *q_i_* are the binding interactions in each structure and *P* = {*p_i_*} and *Q* = {*q_i_*}.

##### Grid Heatmaps

A heat maps is a 2-dimensional data visualization technique, where the magnitude of individual values within a dataset is color coded, i.e., represented by a color. It helps capturing the most relevant data. In the biological field, heat maps are used to visually represent patterns in DNA, RNA or gene expression.

We applied grid color coded heat maps to visualize binding modes of the ligands in the solid state and when docked to the active pocket in dCK. In both cases, a red-yellow-blue scheme was used, where dark red indicates strong interactions and dark blue indicates very weak interactions. Additionally, heat maps were used to visualize differences in the binding modes in the solid state and protein–ligand complex (the native ligand was treated as a reference). In this case, the diagram in red-yellow-blue scheme shows the strengthening (red), stability (yellow) and weakening (blue) of the binding strength.

## 4. Conclusions

A slight structural alteration, the inversion of hydroxyl group at the 2′-position of the sugar, leads to change of the biological activity from anti-depressant and DNA/RNA block builder (cytidine) to powerful anti-cancer (Ara-C). We have discovered that the abovementioned modification causes significant changes in the electron density distribution and binding mode, clearly visible in the NQR spectrum.

This butterfly effect in Ara-C can be seen in the individual molecules, solid state but also after the docking to the active pocket in dCK. To evaluate it, we proposed the new parameter, the root-mean-standard deviation of the binding modes and heat maps approach. They allow to compare binding modes in crystals and protein–ligand complexes. The root-mean-square deviation of the binding mode RMSD_BM defined by us enables the assessment of global differences in the binding mode in solid state and protein–ligand complex. The heat map of binding strength for each ligand versus residue allows identification of the strongest and most stable components of the binding mode. Differential heat maps make it easier to assess which residues are important for binding efficiency.

The relative reactivity, R^+^ and R^−^, acts as ligand screening parameters: smaller R values means stronger binding to dCK. Using the abovementioned techniques, we identified the intramolecular OH···O hydrogen bond as the key factor responsible for forcing the glycone conformation and strengthening NH···O bonds with Gln97, Asp133, and Ara128, and stacking with Phe137. The title butterfly effect is associated with intramolecular hydrogen bond. Our study elucidates the differences in the binding modes of Ara-C and cytidine, which should guide the design of more potent anti-cancer and anti-viral analogues.

## Figures and Tables

**Table 1:** ^14^N NQR frequencies, quadrupole coupling constants e^2^qQ/h and the asymmetry parameters η in Ara-C at T = 295 K and related compounds.

Compound	Nitrogen Site	ν_+ _ [MHz]	ν_−_ [MHz]	ν_0_ [MHz]	|e^2^qQ/h| [MHz]	η	Assignment
Ara-C	N(1)	2.555	2.055	0.500	3.073	0.325	–NH_2_
	N(2)	2.890	1.800	1.090 *	3.147	0.712	–N=
	N(3)	2.530	2.315	0.215	3.230	0.133	>N-sugar
cytidine ^1^	N(1)	2.476	1.974	0.502	2.967	0.338	–NH_2_
	N(2)	2.683	1.463	1.220	2.769	0.883	–N=
	N(3)	2.356	-	-	-	-	>N-sugar
5-azacytidine ^1^	N(1)	2.140	1.420	0.715	2.373	0.603	–NH_2_
	N(2)	2.740	2.120 *	0.620	3.240	0.383	–N(3)= ^1^
	N(3)	2.720	2.100 *	0.620	3.243	0.386	–N(5)= ^1^
	N(4)	2.200	-	-	-	-	>N-sugar ^1^
cytosine ^1,2^	N(1)	2.492	1.922	0.570	2.491	0.388	–NH_2_
	N(2)	2.723	1.570	1.153	2.862	0.806	–N=
	N(3)	2.028	1.219	0.801	2.165	0.740	>NH

NQR frequencies from: ^1^ ref [27], ^2^ ref [28,29]. * calculated from ν_+_ − ν_−_ = ν_0_.

**Table 2:** The ^14^N NQR parameters for Ara-C and related compounds calculated theoretically at the GGA/RPBE level with TS DFT-D correction.

Structure	Site	ν_+_ [MHz]	ν_−_ [MHz]	ν_0_ [MHz]	e^2^qQ/h [MHz]	η	s, r^2^
Ara-C ^1^ hydrogens optimized	–NH_2_	2.658	2.175	0.483	−3.222	0.30	0.065, 0.997
	–N=	2.835	1.970	0.865	−3.203	0.54	
	>N-sugar	2.565	2.289	0.275	−3.236	0.17	
Ara-C ^1^ X-ray hydrogens	–NH_2_	2.568	1.677	0.891	−2.83	0.63	
	–N=	2.844	2.031	0.813	−3.25	0.50	0.235, 0.940
	>N-sugar	2.519	2.218	0.300	−3.158	0.19	
β-cytidine ^2^ hydrogens optimized	–NH_2_	2.566	2.276	0.291	−3.228	0.18	0.158, 0.974
	–N=	2.699	1.619	1.080	−2.879	0.75	
	>N-sugar	2.406	2.221	0.185	−3.085	0.12	
β-cytidine ^2^ X-ray hydrogens	–NH_2_	2.506	1.718	0.788	−2.816	0.56	
	–N=	2.709	1.744	0.965	−2.969	0.65	0.229, 0.921
	>N-sugar	2.335	2.113	0.222	−2.965	0.15	
2′-deoxycytidine form β ^3^ hydrogens optimized	–NH_2_	2.432	1.702	0.730	−2.756	0.53	-
	–N=	2.744	1.868	0.876	−3075	0.57	
	>N-sugar	2.361	2.079	0.281	−2.960	0.19	
3′-deoxycytidine ^4^ hydrogens optimized	–NH_2_	3.267	2.481	0.786	−3.832	0.41	unreliable result due to poor quality structure
	–NH_2_	4.473	4.020	0.453	−5.662	0.16	
	–N=	2.741	1.728	1.013	−2.979	0.68	
	–N=	2.945	2.298	0.647	−3.495	0.37	
	>N-sugar	2.582	2.275	0.308	−3.238	0.19	
	>N-sugar	2.201	1.965	0.236	−2.777	0.17	
3′-deoxycytidine single molecule optimized geometry	–NH_2_	3.178	2.876	0.303	−4.036	0.15	-
	–N=	2.958	2.216	0.742	−3.449	0.43	
	>N-sugar	2.373	2.076	0.297	−2.966	0.2	
2′,3′-dideoxycytidine (Zalcitabine) ^5^ hydrogens optimized	–NH_2_	2.525	2.151	0.374	−3.117	0.24	-
	–N=	2.673	1.627	1.046	−2.867	0.73	
	>N-sugar	2.317	2.153	0.164	−2.980	0.11	
5-azacytidine (Vidaza) structure predicted	–NH_2_	2.621	2.188	0.433	−3.206	0.27	0.439, 0.832
	–N(3)=	2.609	1.415	1.194	−2.683	0.89	
	–N(5)=	2.842	2.453	0.388	−3.530	0.22	
	>N-sugar	2.080	1.985	0.095	−2.710	0.07	
5-azacytidine (Vidaza) geometry optimized single molecule	–NH_2_	3.072	2.779	0.293	−3.901	0.15	0.548, 0.763
	–N(3)=	2.932	2.094	0.838	−3.351	0.50	
	–N(5)=	2.763	2.338	0.425	−3.401	0.25	
	>N-sugar	2.316	1.882	0.434	−2.799	0.31	
cytosine ^6^ hydrogens optimized	–NH_2_	2.732	2.005	0.726	−3.158	0.46	0.175, 0.955
	–N=	2.643	1.643	1.000	−2.857	0.70	
	>NH	2.243	1.537	0.706	−2.520	0.56	

X-ray structures from ^1^ ref. [30], ^2^ ref. [31], ^3^ ref. [32], ^4^ ref. [33], ^5^ ref. [34], ^6^ ref. [35].

**Table 3:** Percentage contributions to the 3D Hirshfeld surface area calculated for each pair of species.

Compound	Structure	Homonuclear				Heteronuclear					
		CC	HH	NN	OO	CH/HC	CN/NC	CO/OC	NH/HN	OH/HO	NO/ON
Ara-C	6.4%, 295 K [30]	0	41.5	0	0.2	8.8	1.0	4.1	12	31.2	2.6
α-cytidine	3.3%, 295 K [38]	2.8	48.7	0.7	1.2	3.3	1.7	1.9	8.7	29.3	1.8
β-cytidine	2.87%, 295 K [39]	0.2	44.3	0.3	0.5	7.6	1.0	3.8	9.7	30.6	2.1
	2.32%, 297.2 K [31]	0.1	44.8	0.4	0.4	7.9	0.6	2.0	10.1	30.0	2.1
	4.57%, 2 K [40]	0.1	44.6	0.5	0.2	7.8	0.8	4.1	9.8	30.0	2.2
	9.88%, 293 K [35]	0.8	42.5	0.6	0.4	6.9	0.9	4.0	9.9	32.0	2.0
	5.6%, 295 K [41]	0.2	44.2	0.5	0.4	8.1	0.9	3.5	10.2	30.2	1.9
2′-deoxycytidine, α	3.65%, 100 K [42]	1.0	44.2	0	0	9.5	0.3	0.1	13.4	31.2	0.3
2′-deoxycytidine, β	5.4%, 295 K [32]	0	43.4	0	0.1	7.8	0.0	1.2	13	33.8	0.7
3′-deoxycytidine	7.3%, 295 K [33]	1.7	45.2	0	2.7	8.9	1.2	0.5	10.2	26.0	3.7
2′,3′-dideoxycytidine (Zalcitabine)	5%, 295 K [43]	0	48.7	0	0.5	12.6	0	0.9	13.7	22.0	0.8
	3.7%, 295 K [34]	0	49	0	0.4	12.6	0.0	0.8	13.9	22.5	0.8
5-aza-2′-deoxycytidine (Decitabine)	2.96% 150 K [44]	0	42.8	0.9	0.5	6.1	1.6	2.2	17.8	25.0	3.2
cytosine, form I	7.0%, 295 K [45]	2.9	31.7	0.3	0.1	11.2	5.1	2.3	19.6	25.0	1.7
	3.1%, 295 K [46]	3.3	32.6	0.4	0.1	10.2	5.3	2.3	19.1	25.1	1.6
	0.647%, 293 K [47]	3.7	32.2	0.1	0.2	9.5	5	2.7	20.4	24.2	1.9
cytosine, form II	5.15%, 294 K [48]	1.2	30.5	0.5	0	16.4	0.8	2.6	23.2	22.7	2.2

**Table 4:** The Euclidean distance (ED) and root-mean-square deviation (RMSD) calculated between the 3D Hirshfeld percentage contributions for Ara-C and other compounds studied.

Structure	ED	RMSD
cytidine α	10.58	3.34
cytidine, β	3.95 *	1.25 *
2′-deoxycytidine, α	5.71	1.80
2′-deoxycytidine, β	5.04 *	1.59 *
3′-deoxycytidine	8.21	2.59
2′,3′-dideoxycytidine (Zalcitabine)	12.91 *	4.08 *
5-aza-2′-deoxycytidine (Decitabine)	9.29	2.94
cytosine	14.92 *	4.27 *

* The value averaged over available structures.

**Table 5:** Enrichment ratios E_XY_ characterizing the various contacts in Ara-C and related compounds.

Structure	Atom	C	H	N	O
Ara-C	Surface %	6.95	67.5	7.05	18.4
	C	0	-	-	-
	H	0.94	0.91	-	-
	N	1.02	1.26	0	-
	O	1.60	1.26	0.42	0.06
cytidine α-form	Surface %	6.25	69.3	6.8	17.65
	C	7.17	-	-	-
	H	0.38	1.01	-	-
	N	2.00	0.92	1.51	-
	O	0.86	1.19	0.75	0.39
cytidine β-form	Surface %	6.20	68.25	5.80	17.75
	C	0.26	-	-	-
	H	0.93	0.96	-	-
	N	0.71	1.09	0.87	-
	O	1.68	1.19	0.87	0.13
2′-deoxycytidine α-form	Surface %	5.9	71.27	7	15.77
	C	2.87	-	-	-
	H	1.13	0.87	-	-
	N	0.36	1.34	0.00	-
	O	0.00	1.39	0.14	0.00
2′-deoxycytidine β-form	Surface %	4.5	70.7	6.8	17.9
	C	0.00	-	-	-
	H	1.23	0.87	-	-
	N	0.00	1.35	0.00	-
	O	0.74	1.34	0.25	0.03
3′-deoxycytidine	Surface %	7	67.8	7.6	17.8
	C	3.47	-	-	-
	H	0.94	0.98	-	-
	N	1.14	1.00	0.00	-
	O	0.20	1.08	1.38	0.85
	Surface %	6.7	73.5	7.3	12.5
Zalcitabine	C	0.00	-	-	-
	H	1.28	0.91	-	-
	N	0.00	1.29	0.44	-
	O	0.48	1.23	0.26	0.00
	Surface %	4.95	67.25	12.2	15.7
	C	0.00	-	-	-
Decitabine	H	0.92	0.95	-	-
	N	1.32	1.08	0.60	-
	O	1.42	1.18	0.84	0.20
cytosine	Surface %	12.3	59.25	13.8	14.65
	C	2.45	-	-	-
	H	0.65	0.92	-	-
	N	1.47	1.25	0.05	-
	O	0.75	1.39	0.49	0.09

**Table 6:** The Euclidean distance (ED) and root-mean-square deviation (RMSD) between the enrichment ratios for Ara-C and related compounds.

Structure	ED	RMSD
cytidine α	7.33 (1.58 *)	1.46 (0.26 *)
cytidine, β	1.02	0.17
2′-deoxycytidine, α	2.93	0.49
2′-deoxycytidine, β	0.60	0.10
3′-deoxycytidine	3.48	0.58
2′,3′-dideoxycytidine (Zalcitabine)	0.74	0.12
5-aza-2′-deoxycytidine (Decitabine)	0.72	0.12
cytosine	2.50	0.42

* Excess value for CC contacts omitted.

**Table 7:** Key hydrogen bonding interactions in the solid state (E_tot_—total energy, E_e_—electrostatic term, E_P_—polarization term, E_d_—dispersion and E_r_—repulsion).

Compound	Hydrogen Bond	R_A∙∙∙B_ [Å]	<AHB [°]	E_e_ [kJ/mol]	E_p_ [kJ/mol]	E_d_ [kJ/mol]	E_r_ [kJ/mol]	E_tot_ [kJ/mol]	Moiety
Ara-C	OH∙∙∙O *	2.650	155.45	-	-	-	-	-	Ry
	CH∙∙∙O *	3.685	157.20	-	-	-	-	-	Ry
	NH∙∙∙O	2.983	163.70	−32.7	−8.9	−21.1	28.1	−42.3	–NH_2_
	NH∙∙∙O	3.048	145.73	−16.3	−10.2	−27	29	−30.4	–NH_2_
	OH∙∙∙O	3.003	159.64	−33.4	−9.3	−23.4	30.5	−43.7	Ry
	OH∙∙∙O	2.720	171.72	−74.7	−25.5	−20.5	66.6	−74.5	Ry
	CH∙∙∙N	3.652	159.76						–N=
Cytidine α-form	CH∙∙∙O *	2.662	101.87	-	-	-	-	-	C(6)H/Ry
	CH∙∙∙O *	2.716	98.15	-	-	-	-	-	=O/Ry
	NH∙∙∙O	2.946	171.58	−28.1	−6.6	−12.6	29.2	−27.5	–NH_2_
	NH∙∙∙O	3.183	144.97	−30.4	−8.7	−15	18.7	−40.0	–NH_2_
	CH∙∙∙O	3.292	141.69						=O
	OH∙∙∙O	2.674	151.65	−45.3	−9.3	−13.1	49.6	−35.6	Ry
	OH∙∙∙N	2.752	168.40	−132.5	−38.9	−57	169.4	−113.9	–N=
	π···π stacking	3.473	-						rings
Cytidine β-form	CH∙∙∙O *	3.233	151.50	-	-	-	-	-	Ry
	CH∙∙∙O *	2.676	92.10	-	-	-	-	-	=O/Ry
	CH∙∙∙O *	2.347	97.60	-	-	-	-	-	C(6)H/Ry
	NH∙∙∙O	2.946	147.84	−54.4	−10.5	−15.3	43.4	−51.8	–NH_2_
	NH∙∙∙O	2.923	145.81						–NH_2_
	OH∙∙∙N	2.860	174.31	−114.8	−35.9	−24.6	115.0	−98.3	–N=
	OH∙∙∙O	2.711	174.79						Ry
	CH∙∙∙O	3.346	152.44	1.9	−2.9	−11.2	12.2	−2.4	C(5)/OH(Ry)
2′-deoxycytidine α form	CH∙∙∙O *	2.689	85.46	-	-	-	-	-	=O
	OH∙∙∙O	2.716	150.58	−92.2	−27	−26.6	89	−86.4	=O
	NH∙∙∙O	2.937	157.77						–NH_2_
	CH∙∙∙O	3.317	142.59						Ry
	NH∙∙∙O	2.949	167.22	−33.3	−8.9	−31.8	47.7	−40.0	–NH_2_
2′-deoxycytidine β form	CH∙∙∙HO	3.040	98.37 127.10	-	-	-	-	-	-
	CH∙∙∙O	2.716	103.73	-	-	-	-	-	=O/Ry
	CH∙∙∙O	3.384	157.63	-	-	-	-	-	-
	NH∙∙∙O	2.967	141.59	−83.2	−20.3	−17.2	64.3	−78.3	–NH_2_
	NH∙∙∙N	3.018	156.22						–N=
	NH∙∙∙O	3.009	153.35	−49.7	−15.3	−23.8	52.8	−52.0	–NH_2_
	OH∙∙∙O	3.153	137.88						Ry
3′-deoxycytidine	CH∙∙∙O *	2.753	111.88	-	-	-	-	-	C(6)H/Ry
	CH∙∙∙O *	2.687	95.40	-	-	-	-	-	=O/Ry
	OH∙∙∙HO	2.820	117.07	−5.0	−17.3	−14.3	58.2	5.5	Ry
	NH∙∙∙O	2.858	143.47	−39.8	−11.2	−14.2	35.6	−40.7	–NH_2_
	NH∙∙∙O	3.503	144.35	−0.1	−10	−16.9	29.5	−4	–NH_2_
Zalcitabine	CH∙∙∙O *	3.226	168.19	-	-	-	-	-	Ry
	CH∙∙∙O *	2.726	95.53	-	-	-	-	-	C(6)H/Ry
	CH∙∙∙O *	2.682	95.91	-	-	-	-	-	=O/Ry
	NH∙∙∙O	2.977	166.77	−37	−11.6	−13.7	30	−41.2	–NH_2_
	NH∙∙∙O	3.268	159.33	−24.8	−4.8	−15.7	15.1	−34.2	–NH_2_
	OH∙∙∙N	2.774	168.64	−73.5	−23.3	−26.4	75.3	−62.8	–N=
Decitabine	CH∙∙∙O *	2.687	98.61	-	-	-	-	-	C(6)H/Ry
	NH∙∙∙N	2.873	166.70	−89.2	−22.9	−18.7	104.8	−62.9	–NH_2_
	OH∙∙∙O	2.678	170.94	−52.5	−15.8	−16.2	60.0	−44.2	=O
	OH∙∙∙O	2.742	153.01	−38.4	−9.1	−23.2	51.6	−35.7	Ry
Cytosine	NH∙∙∙O	3.022	166.42	−101.4	−27	−17.5	84.8	−90.1	–NH_2_
	NH∙∙∙N	2.791	144.49	−41.2	−10.8	−5.6	23.5	−42.9	–N=
	NH∙∙∙O	2.991	165.21						=O
	π···π stacking	3.81	-	20.2	−5/5	−26.1	14.3	3.3	-

* intramolecular hydrogen bond.

**Table 8:** Differences in the binding modes between Ara-C and the studied compounds in solid state.

Compound		RMSD_BM			
	Total	HH	OH	NH	CH
cytidine α	0.1385	0.1226	0.1057	0.0989	0.0967
cytidine, β	0.1217	0.1177	0.1040	0.1029	0.0900
2′-deoxycytidine, α	0.1130	0.1011	0.1089	0.1040	0.0987
2′-deoxycytidine, β	0.1255	0.1062	0.1088	0.1115	0.0998
3′-deoxycytidine	0.1267	0.1118	0.1088	0.1052	0.0991
2′,3′-dideoxycytidine (Zalcitabine)	0.1165	0.1080	0.1098	0.1040	0.0955
5-aza-2′-deoxycytidine (Decitabine)	0.1161	0.1046	0.1055	0.1001	0.0865
cytosine	0.1307	0.1143	0.1138	0.1056	0.0931

**Table 9:** The docking results for the Ara-C, cytidine and related compounds; kcal/mol units.

Parameter	Ara-C	2′-deoxycytidine, 1P60	2′-deoxycytidine, 1P61	2′-deoxycytidine	cytidine	Vidaza (5-azacytidine)	3′-deoxycytidine	Zalcitabine (2′-3′-dideoxycytidine)	3′-dehydroxy, 3,4′-didehydrocytidine	Gemcitabine, (2′, 2′-difluoro, 2′deoxycytidine), 1P62	Gemcitabine (2′, 2′-difluoro,2′deoxycytidine)	Decitabine (5-aza-2′-deoxycytidine)
Total energy	−105.083	−101.556	−110.763	−99.252	−94.384	−99.241	−101.853	−96.519	−102.211	−96.221	−100.881	−96.171
Protein-ligand	−120.891	−114.636	−114.816	−111.000	−105.813	−107.250	−109.095	−103.851	−101.898	−116.96	−121.854	−108.103
Steric	−103.105	−100.597	−101.318	−97.634	−97.468	−95.089	−97.641	−94.237	95.288	−102.44	−104.982	−93.800
Hydrogen bonding	−19.582	−14.040	−13.498	−15.221	−12.397	−12.667	−12.498	−10.000	−11.310	−14.821	−16.872	−14.299
Mg^2+^-ligand	−0.149	0	0	−0.047	−0.300	−0.225	−0.176	−0.073	−0.313	−0.094	−0.153	−0.049
Binding affinity *	−25.40	−23.07	−23.62	−24.58	−21.82	−21.30	−22.37	−24.18	−22.16	−30.76	−31.56	−23.62
Binding affinity **	−6.90	−6.84	−6.75	−6.81	−6.87	−6.06	−6.84	−6.70	−6.79	−7.54	−7.33	−6.30

* Gehlhaar model, kJ/mol, ** PRODIGY model, kcal/mol.

**Table 10:** The binding mode of the Ara-C, cytidine and its analogues to dCK (the residues are ordered by decreasing binding energy expressed in kcal/mol).

Residue	Ara-C 15PZ	Ara-C	2′-deoxycytidine, 1P60	2′-deoxycytidine, 1P61	2′-deoxycytidine	Cytidine	5-azacytidine	3′-deoxycytidine	Zalcitabine	3′-dehydroxy, 3,4′-didehydrocytidine	Gemcitabine, 1P62	Gemcitabine	Decitabine
Phe137	−23.73	−24.86	−22.425	−24.102	−24.51	−21.75	−23.16	−23.97	−23.52	−22.668	−26.063	−25.343	−19.972
Gln97	−10.46	−10.55	−9.412	−10.040	−10.67	−7.77	−8.51	−10.58	−10.91	−7.796	−10.135	−11.251	−10.409
Glu53	−9.87	−10.53	−8.544	−6.823	−9.00	−9.34	−9.68	−10.89	−8.61	−9.036	−10.741	−11.348	−8.230
Phe96	−10.30	−10.03	−10.662	−10.039	−9.60	−10.57	−10.71	−10.98	−11.17	−9.935	−9.887	−9.895	−11.919
Arg128	−10.32	−9.83	−8.098	−8.338	−9.55	−6.34	−5.62	−5.95	−5.61	−6.320	−6.922	−8.340	−6.112
Trp58	−8.89	−9.15	−10.177	−10.736	−7.17	−10.73	−9.18	−10.52	−8.59	−10.828	−8.875	−9.480	−10.151
Asp133	−7.89	−8.05	−7.168	−7.856	−6.42	−4.07	−6.89	−8.08	−7.76	−4.878	−7.972	−8.040	−7.635
Ile30	−7.03	−7.19	−6.473	−5.779	−6.80	−6.43	−4.45	−2.43	−4.16	−4.224	−6.875	−7.546	−4.279
Tyr86	−7.50	−7.07	−8.099	−7.299	−5.50	−7.42	−7.18	−6.02	−4.28	−6.2	−5.787	−4.994	−7.296
Glu197	−3.19	−6.44	−2.983	−5.422	−6.16	−5.16	−5.50	−2.56	−2.75	−3.092	−5.961	−6.378	−3.423
Leu82	−5.34	−5.03	−6.464	−6.028	−2.02	−5.05	−4.37	−4.37	−3.96	−4.592	−5.804	−5.372	−6.131
Val55	−2.40	−2.71	−2.405	−3.650	−1.65	−3.55	−3.62	−3.13	−2.95	−4.163	2.626	−2.361	−2.248
Met85	−2.03	−2.01	−1.965	−1.903	−2.02	−1.61	−3.62	−3.13	−2.95	−1.950	−1.864	−2.002	−2.471
Ala100	−1.53	−1.66	−1.467	−1.367	−1.29	−1.29	−1.49	−1.49	−1.65	−1.524	−1.418	−1.858	−1.955
Arg104	−1.31	−1.38	−1.359	−1.219	−1.26	−1.21	−0.96	−1.39	−1.30	−1.387	−1.365	−1.744	−0.895
Leu141	0.00	0.00	0	0	−0.36	0.00	0.00	−0.34	−0.47	0	0	0	−0.330
Arg194	0.00	0.00	−0.405	0	0.00	−0.33	0.00	0.00	0.00	0	0	0	0
Ile200	−0.48	−0.46	0	−0.47	−0.52	−0.33	−0.31	0.00	0.00	0	−0.573	−0.539	−0.420

**Table 11:** The root-mean-square deviation of binding mode (RMSD_BM).

Residue	Ara-C, 1P5Z	Ara-C	2′-deoxycytidine, 1P60	2′-deoxycytidine, 1P61	2′-deoxycytidine	cytidine	5-azacytidine	3′-deoxycytidine	Zalcitabine	3′-dehydro-3,4-dideoxycytitine	Gemcitabine, 1P62	Gemcitabine	Decitabine
RMSD_BM	-	0.878	-	-	1.378	1.739	1.622	1.717	1.666	1.738	-	1.259	1.230
RMSD_BM *	-	0.878	0.913	1.230	-	-	-	-	-	-	1.786	-	-
RMSD_BM **	-	0.878	1.933	1.660	-	-	-	-	-	-	1.354	-	-
RMSD_BM ***	-	-	0.989	0.989	-	-	-	-	-	-	-	-	-

* Binding mode distance between Ara-C and the native ligand. ** Binding mode distance between the original and redocked ligand. *** Binding mode distance between the redocked ligands of 2′-deoxycytidine.

**Table 12:** Identification of hydrogen bonds crucial for the ligand-protein bond.

Residue	Ara-C	2′-deoxycytidine	Cytidine	5-azacytidine	3′-deoxycytidine	Zalcitabine	3′deoxy-3′,4′-didehydrocytidine	Gemcitabine	Decitabine
Phe137	π···π	π···π	π···π	π···π	π···π	π···π	π···π	π···π	π···π
	hydrophobic	hydrophobic	hydrophobic	hydrophobic	hydrophobic	hydrophobic	hydrophobic	hydrophobic	hydrophobic
Gln97	–NH_2,_ –N=	–NH_2,_ –N=	–NH_2,_ –N=	–N=	–NH_2_	–NH_2,_ –N=	–NH_2,_ –N=	–NH_2,_ –N=	–NH_2,_ –N=
Glu53	CH_2_OH	CH_2_OH	CH_2_OH	CH_2_OH	CH_2_OH	CH_2_OH	CH_2_OH	CH_2_OH	CH_2_OH
Phe96	hydrophobic	hydrophobic	hydrophobic	hydrophobic	hydrophobic	hydrophobic	hydrophobic	hydrophobic	hydrophobic
Arg128	OH, CH_2_OH	CH_2_OH	-	-	-	-	-	CH_2_OH	CH_2_OH
Trp58	-	-	-	-	-	-	-	-	-
Asp133	NH_2_	NH_2_	NH_2_	NH_2_	NH_2_	NH_2_	NH_2_	NH_2_	NH_2,_
Ile30	-	-	-	-	-	-	-	-	-
Tyr86	OH	OH	OH	OH	OH	-	OH	OH	OH
Glu197	OH	OH	OH	OH	-	-	-	OH	-

**Table 13:** The angle describing the conformations of the ligands in the solid state and protein–ligand complex.

ϕ [°]	Ara-C	2′-deoxycytidine 1P60	2′-deoxycytidine 1P61	2′-deoxycytidine	Cytidine	Cytidine	5-azacytidine	3′-deoxycytidine	Zalcitabine	3′dehydro-3,4′-dideoxy	Gemcitabine, 1P62	Gemcitabine	Decitabine
solid state	28.83	-	-	44.14	-	18.30	-	35.64	23.29	-	-	-	18.13
protein-ligand	36.41	49.63	41.42	47.99	55.69	46.52	48.48	50.29	66.43	48.05	47.59	38.30	18.13

**Table 14:** Theoretical global indices: absolute electronegativity, χ; absolute hardness, η; electrophilicity index (reactivity), ω; softness, S; electro-donating power, ω-; electro-accepting power ω+; net electrophilicity, Δω; relative electro-donating electrophilicity R^+^ and relative electro-accepting electrophilicity R^−^ calculated for the ligands studied at the M062X/6-311G(d,p) level of the theory.

Ligand	HOMO [eV]	LUMO [eV]	Gap [eV]	Χ [eV]	η [eV]	ω [eV]	S [1/eV]	ω^+^ [eV]	ω^− ^ [eV]	Δω [eV]	R^+ ^ [−]	R^− ^ [−]
Ara-C *	−7.490	0.143	7.633	3.673	3.816	1.768	0.262	4.082	0.408	4.490	1.000	1.000
Ara-C	−7.321	0.204	7.525	3.559	3.762	1.683	0.266	3.933	0.374	4.307	0.964	0.916
cytidine	−7.707	−0.748	6.959	4.228	3.479	2.568	0.287	5.117	0.890	6.007	1.254	2.179
2′-deoxycytidine	−7.308	0.151	7.458	3.578	3.729	1.717	0.268	3.972	0.394	4.366	0.973	0.965
3′-deoxycytidine	−7.709	−0.208	7.501	3.958	3.750	2.089	0.267	4.537	0.579	5.115	1.112	1.417
Zalcitabine	−7.549	0.079	7.628	3.735	3.814	1.828	0.262	4.173	0.438	4.611	1.022	1.072
5-azacytidine	−8.406	−0.488	7.917	4.447	3.959	2.498	0.253	5.216	0.769	5.985	1.278	1.884
Gemcitabine	−7.122	0.107	7.229	3.507	3.614	1.702	0.277	3.907	0.400	4.307	0.957	0.979
Decitabine	−7.983	0.187	8.170	3.898	4.085	1.859	0.245	4.319	0.421	4.740	1.058	1.032

* native ligand

**Table 15:** The spectrum, of the biological activity.

	Ara-C	2′-deoxycytidine	cytidine	Vidaza	3′-deoxycytidine	Zalcitabine	3′-dehydroxy, 3,4′-didehydrocytidine	Gemcitabine	Decitabine
Biological activity	anti-leukemic (DNA, RNA)	DNA component	RNA component	anti-leukemic (RNA, DNA)	anti-virial, anticancer (DNA)	anti-retroviral	anti-virial	anti-neoplastic, anti-cancer, anti-tumor anti-virial	anti-leukemic (DNA)
Spectrum of activity *	AML, ALL CML	natural	natural, anti-depressant	MDS, JMML	NHL	HIV/ AIDS	metabolite	HEV, anti-cancer (pancreatic, ovarian, non-small cell lung	MDS, AML, HPV

* AML (Acute myeloid leukemia), ALL (Acute lymphoblastic leukemia), CML (chronic myeloid leukemia), MDS (Myelinoclastic diffuse sclerosis), JMML (Juvenile myelomonocytic leukemia), NHL (non-Hodgkin lymphoma), HIV/AIDS (Human immunodeficiency virus infection and acquired immunodeficiency syndrome), HEV (Hepatitis E virus), HPV (Human papilloma virus).

## Data Availability

No new data were created or analyzed in this study. Data sharing is not applicable to this article.
